# Restarted replication forks are error-prone and cause CAG repeat expansions and contractions

**DOI:** 10.1371/journal.pgen.1009863

**Published:** 2021-10-21

**Authors:** Michaela A. Gold, Jenna M. Whalen, Karine Freon, Zixin Hong, Ismail Iraqui, Sarah A. E. Lambert, Catherine H. Freudenreich

**Affiliations:** 1 Department of Biology, Tufts University, Medford, Massachusetts, United States of America; 2 Institut Curie, Université PSL, Orsay, France; 3 Université Paris-Saclay, Orsay, France; 4 Equipes Labélisées Ligue Nationale Contre Le Cancer, Orsay, France; The Hospital for Sick Children and University of Toronto, CANADA

## Abstract

Disease-associated trinucleotide repeats form secondary DNA structures that interfere with replication and repair. Replication has been implicated as a mechanism that can cause repeat expansions and contractions. However, because structure-forming repeats are also replication barriers, it has been unclear whether the instability occurs due to slippage during normal replication progression through the repeat, slippage or misalignment at a replication stall caused by the repeat, or during subsequent replication of the repeat by a restarted fork that has altered properties. In this study, we have specifically addressed the fidelity of a restarted fork as it replicates through a CAG/CTG repeat tract and its effect on repeat instability. To do this, we used a well-characterized site-specific replication fork barrier (RFB) system in fission yeast that creates an inducible and highly efficient stall that is known to restart by recombination-dependent replication (RDR), in combination with long CAG repeat tracts inserted at various distances and orientations with respect to the RFB. We find that replication by the restarted fork exhibits low fidelity through repeat sequences placed 2–7 kb from the RFB, exhibiting elevated levels of Rad52- and Rad8^*Sc*Rad5/*Hs*HLTF^-dependent instability. CAG expansions and contractions are not elevated to the same degree when the tract is just in front or behind the barrier, suggesting that the long-traveling Polδ-Polδ restarted fork, rather than fork reversal or initial D-loop synthesis through the repeat during stalling and restart, is the greatest source of repeat instability. The switch in replication direction that occurs due to replication from a converging fork while the stalled fork is held at the barrier is also a significant contributor to the repeat instability profile. Our results shed light on a long-standing question of how fork stalling and RDR contribute to expansions and contractions of structure-forming trinucleotide repeats, and reveal that tolerance to replication stress by fork restart comes at the cost of increased instability of repetitive sequences.

## Author summary

Trinucleotide repeat expansions are the cause of several muscular- and neuro-degenerative diseases, and further expansions during intergenerational inheritance often leads to an earlier age-of-onset in the offspring. Some intergenerational expansions appear to occur during germ cell replication, but how those replication-associated expansions arise is incompletely understood. Because there are many replication barriers in genomes, including the structure-forming repeats themselves, we investigated whether a repeat placed after such a barrier had altered instability. We discovered that a restarted replication fork traversing a CAG repeat tract is highly error prone and repeat expansions and contractions are more prevalent in this case compared to normal replication. These results reveal a mechanism for replication-associated repeat instability relevant to disease-associated trinucleotide repeat expansions.

## Introduction

Expanded CAG repeats are responsible for several inherited neurodegenerative diseases including Huntington’s disease (HD), myotonic dystrophy type 1 (DM1) and several types of spinocerebellar ataxia (SCA) [1–2]. CAG/CTG (CAG) repeats form DNA structures, with the CTG strand forming a more stable hairpin than the CAG strand (reviewed in [3]). Hairpin formation during replication or repair can lead to repeat length changes referred to as repeat instability, including expansions and contractions [4–5]. Disease-causing repeat expansions can be quite large, ranging from intermediate lengths of 35 to ~100 repeats at the HD locus, to 100’s-1000’s of repeat units at the DM1 locus [1]. CAG repeat expansions have been shown to occur both in germ cells and somatic cells [6–7]. Instability during non-replicating somatic cells likely occurs during gap repair and is dependent on mismatch repair proteins, primarily Msh2-Msh3 and Mlh1-Mlh3 [8]. Repeat instability during replication may be especially relevant to intergenerational repeat expansions; for example, HD expansions can occur in pre-meiotic replicating testicular germ cells [9].

Compared to expanded CGG or GAA repeats, long CAG repeats are a weak barrier to fork progression as observed by two-dimensional (2D) gel analysis in budding yeast *Saccharomyces cerevisiae* (*S*. *cerevisiae*) [10–11] or by a quantitative PCR-based nascent DNA abundance assay in human cells [12]. However, expanded CAG repeats appear to be especially prone to fork reversal or template switching as visualized by 2D gel electrophoresis and electron microscopy [13–14]. For a CAG tract of about 100 repeats, up to 30% of replication intermediates traversing expanded CAG tracts are converted into joint molecules [11,15], indicating that they are difficult to replicate even if they don’t cause a persistent fork stall. Replication problems at structure-forming repeats can lead to repeat expansions and contractions (reviewed in [16–19]). The lagging strand, which has single-stranded stretches exposed during replication, is particularly prone to DNA structure formation. Defects in polymerization on the lagging strand, for example caused by mutations in Polδ or Polα, cause an increase in contraction frequency of CAG/CTG repeats [20–21]. An established mechanism for repeat expansions is structure formation on the displaced 5’ flap of the Okazaki fragment, which renders it resistant to Fen1 cleavage; ligation of the unprocessed flap due to flap equilibration incorporates the additional sequence, leading to an expansion (reviewed in [22–23]). Another response to lagging strand hairpins can be a template switch to copy from the sister chromatid, which has been shown by multiple groups to cause repeat expansions (see [17] for review). It is less clear how often structures occur during leading strand replication, where Polε is tightly coupled to the MCM helicase. However, a pre-formed structure could cause fork uncoupling or fork reversal. A third proposed mechanism for expansions is formation of a hairpin on the reversed leading strand which is subsequently incorporated during fork restart [17,24]. Recent results show that a late S-phase event that results in accumulation of proteins known to bind to collapsed replication forks happens at an expanded (CAG)130 repeat and provokes relocation to the nuclear pore complex to limit repeat instability [25–26].

Despite the abundant evidence that repeat instability occurs during replication of structure-forming trinucleotide repeats, it is still unclear whether these events occur during passage of the replication fork through the repeat during normal replication or only after a repeat-dependent replication fork stalling event that requires fork restart at the repeat-induced barrier. And for the restart model, it is unknown whether repeat instability would be more likely to occur during the initial restart process or during the progression of the restarted fork through the remainder of the repeat tract. To separate out these events, we utilized an established and inducible replication fork barrier (RFB) system on fission yeast *Schizosaccharomyces pombe* (*S*. *pombe*) chromosome 3 and cloned a (CAG/CTG)70 repeat at various distances and orientations from the barrier. In this system, recombination-dependent fork restart has been established to occur by a Rad52-dependent mechanism that does not involve a broken fork intermediate [27–28]. Replication by the restarted fork, termed recombination-dependent replication (RDR), was shown to be liable to ectopic recombination, template switches, and replication slippage [27,29–31]. In this way, we were able to create a strong fork stall independent of the repeat tract and measure the effect of fork restart through the repeat tract. We show that a restarted fork traversing through the expanded CAG tract is highly mutagenic, resulting in an increased frequency of expansions and contractions. Surprisingly however, the greatest instability did not occur during the initial restart process, but during replication by the established restarted fork which utilizes Polδ for both leading and lagging strand replication (δ-δ fork) [32]. In addition, a switch in replication direction, which occurred due to the fork barrier causing the repeat to be replicated by a converging fork, caused additional repeat instability. Our results indicate that restarted forks are highly prone to replication slippage mistakes within repetitive DNA, which can lead to repeat length changes similar to those observed to cause repeat expansion diseases.

## Results

### CAG-70 repeat instability occurs during replication fork restart after induction of a replication fork barrier (RFB)

**Fig 1:**
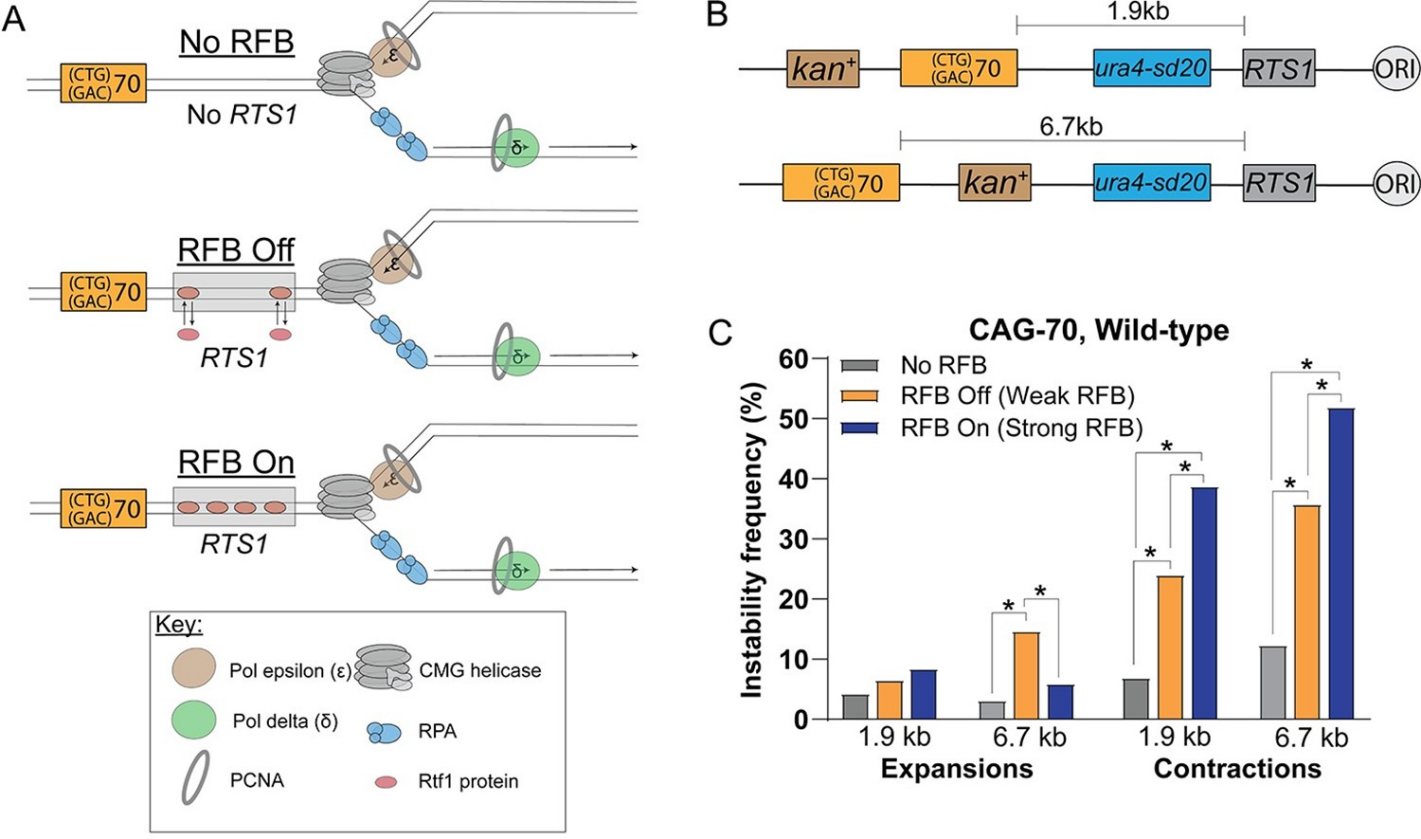
Increased CAG-70 repeat instability upon induction of a replication fork barrier (RFB) 1.9 Kb or 6.7 Kb upstream from the CAG repeats. A) The three conditions tested in the replication fork barrier (RFB) fork restart assay. The Rtf1 protein is red and the *RTS1* sequence is indicated by the gray box. In the No RFB condition there is no *RTS1* sequence such that no RFB can form. In the RFB Off/Weak RFB condition one or two Rtf1 proteins may bind transiently to the *RTS1* sequence due to slightly leaky repression of Rtf1 expression by addition of thiamine. In the RFB On/Strong RFB condition Rtf1 proteins will fully occupy the 4 binding sites present in the *RTS1* sequence as the Rtf1 protein is fully expressed without the addition of thiamine. B) Schematic of the CAG-70 repeat location in relation to the *RTS1* sequence. CAG-70 repeats were integrated such that CAG was on the lagging strand template either 1.9 kb or 6.7 kb downstream of the *RTS1* sequence. (C) Percent contractions and expansions of the CAG-70 repeat after RFB induction for wild-type strains at both the 1.9 kb and 6.7 kb locations across conditions as indicated. (*) p≤0.05 compared to No RFB or Weak RFB (as indicated by brackets) by Fisher’s Exact Test. If no star is present, it indicates that the comparisons showed no significant difference. See Table A in S1 Text for exact number of colonies analyzed (n = 109–487) and exact percentages for individual assays. See Table K in S1 Text for P-values.

In order to test the effect of RDR on CAG repeat instability, 70 CAG repeats (CAG-70) were integrated 1.9 kb or 6.7 kb downstream from the *RTS1* sequence on *S*. *pombe* chromosome 3, such that the CAG sequence was on the lagging strand template (Figs 1A, 1B and S1A). This orientation was chosen as it is recognized as the more expansion-prone orientation, whereas placement of the CTG sequence on the lagging strand template leads to a high frequency of contractions in *S*. *cerevisiae* [33–35] and human cells [36–37]. The CAG-70 repeat size was chosen as it is long enough to exhibit instability, but not long enough to itself cause a stable replication barrier visible by 2D gel analysis (which occurs in the 100–130 repeat range) [11,15,38]. Indeed, we were not able to detect a stall at the CAG-70 tract integrated into *S*. *pombe* chromosome 3 by 2D gel analysis (S1B Fig). The integration of *RTS1* allows for a single replication fork to be blocked by the binding of several Rtf1 proteins to the *RTS1* sequence to create a polar RFB. The *rtf1*^*+*^ gene is under control of the *nmt41* thiamine repressible promoter, which allows for several conditions to be compared: No RFB (no *RTS1* sequence present), RFB Off (*RTS1* sequence present and Rtf1 protein repressed by addition of thiamine) and RFB On (*RTS1* sequence present and Rtf1 protein expressed, which creates a strong RFB [27] (S1C Fig). After stalling of the replication fork by the RFB, the rate of CAG-70 repeat instability, both expansions and contractions, was assessed by PCR in order to obtain an unbiased view of the instability profile.

In the RFB On condition there was a significant increase in CAG contractions over the No RFB (no *RTS1*) level at both locations (4.2 to 5.6 fold) (Fig 1C). The percent contractions in the RFB On condition was also significantly higher than the RFB Off condition for both the 1.9 kb and the 6.7 kb locations (1.5–1.6 fold) (Fig 1C). Therefore, RFB induction resulted in a dramatic increase in CAG contractions in both locations tested. Unexpectedly, the RFB Off condition also resulted in significantly more CAG contractions than the No RFB condition (2.9 to 3.5 fold). This likely reflects the leakiness of thiamine repression, resulting in some limited binding of the Rts1 protein to the 4 possible binding sites within the *RTS1* sequence and a weak stall, in contrast to the No RFB condition in which the locus is devoid of *RTS1* (Fig 1A). In support of this, 2D gel analysis in the RFB Off condition revealed discrete stalling (S1C Fig) which is further exacerbated in the absence of functional RDR (*i*.*e*. in a *rad52* mutated strain, Fig 1C in [27]). The level of the weak RFB stall is similar to the weak stall observed for a (CAG)100 tract integrated into a *S*. *cerevisiae* chromosome [11,15]. This weak stall in the RFB Off condition was sufficient to cause very mild induction of replication slippage in previous mutational assays (refer to supplementary Fig 1A in [39]). However, CAG contractions are a very frequent event, occurring at a baseline frequency of 7–12% for the 1.9 and 6.7 kb locations respectively, and thus are a very sensitive read-out of replication or repair problems. Based on this data, we refer to the “RFB Off” condition as a “Weak RFB” and “RFB On” as a “Strong RFB” for the purposes of this study. These results indicate that even a weak RFB has a dramatic effect on instability of a neighboring repeat tract, and that the effect is further enhanced when the strength of the barrier is increased.

The CAG-70 expansion frequency baseline was 3–4% at both the 1.9 kb and 6.7 kb locations in the No RFB condition (Fig 1C). Therefore, expansions are less frequent than contractions in *S*. *pombe*, similar to what has been observed in *S*. *cerevisiae*, but also higher than the ~1% expansion frequency observed for this same CAG length, orientation, and methodology at a chromosomal location in *S*. *cerevisiae* [21]. Therefore, trinucleotide repeats (TNRs) are somewhat more unstable in fission yeast compared to budding yeast, similar to the greater expansion frequencies observed in humans and mouse models, and frequent enough to see by a physical PCR assay without selection for mutational events. There was an increase in expansions over the No RFB level for the Weak RFB condition that was highly significant at the 6.7 kb location (p = 0.0008; Fig 1C). Interestingly, the increase in expansions was less in the Strong RFB condition (p = 0.07 compared to No RFB at the 1.9 kb location). This could be partially due to the large increase in contractions occurring in the Strong RFB condition, which is expected to bias against recovery of expansion events. Alternatively, the strong stall may increase the likelihood that the repeat is replicated by the converging (rightward) fork, especially at the 6.7 kb location, resulting in a switch in the direction of replication through the CAG repeat and a reduction in expansions.

We also analyzed the size of both expansion and contraction events in all three conditions (No RFB, Weak RFB, Strong RFB) (S2 Fig). Due to the resolution of the gels used to size the length changes, only changes of +5 or -5 repeats (+15 or -15 bp) or greater were scored, with changes binned in 10 repeat increments. Expansions ranged from about +5 to +50 repeats and contractions ranged from about -5 to -65 repeats. No difference in the range of expansions and contractions among the three conditions was noted. There were, however, significant differences in the distribution of CAG repeat lengths between the no RFB and +RFB conditions by a KS statistical test (p<0.005; Table M in S1 Text). These results indicate that both the level of instability and the distribution of repeat sizes are modified by the presence of an RFB. Overall, we conclude that a replication fork barrier, even a weak or transient one, causes instability at an adjacent CAG repeat tract.

### Rad52-dependent fork restart and Rad8^ScRad5/HsHLTF^-dependent template switch cause CAG-70 repeat expansions

**Fig 2:**
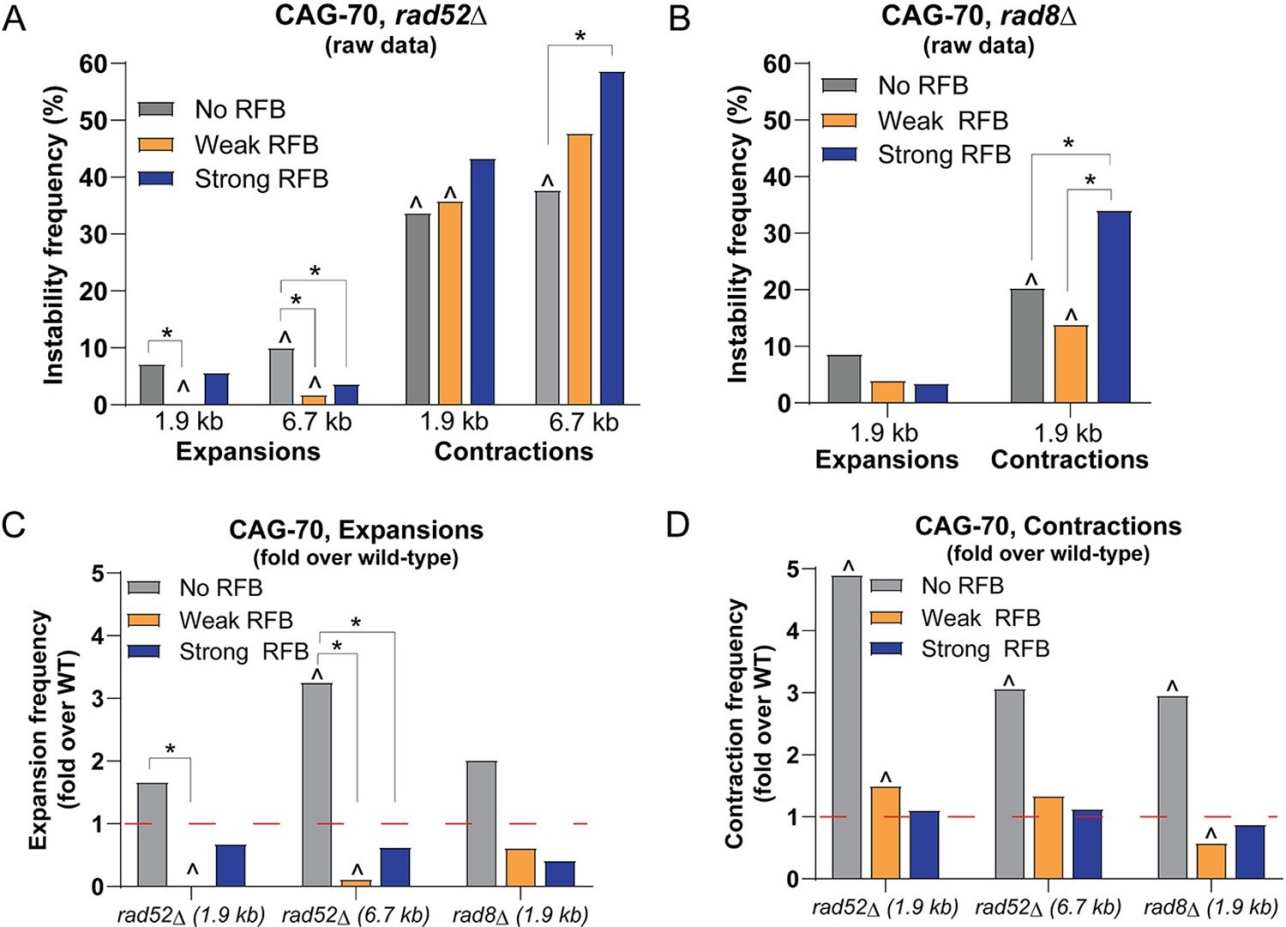
RFB-dependent CAG-70 repeat expansions occur through a Rad52- and Rad8^ScRad5/HsHTLF^-mediated mechanism. A) Percent expansions and contractions for CAG-70 at the 1.9 kb and 6.7 kb locations in a *rad52Δ* mutant across the indicated conditions. B) Percent expansions and contractions for CAG-70 at the 1.9 kb location in a *rad8Δ* mutant across the indicated conditions. (*) p≤0.05 compared to No RFB or Weak RFB (as indicated), (^) p≤0.05 compared to CAG-70 in the wild-type (WT) strain in the same condition by Fisher’s Exact Test. C) Fold over WT percent expansions for CAG-70 repeat in the following mutants: *rad52Δ* at 1.9 kb, *rad52Δ* at 6.7 kb, and *rad8Δ* at 1.9 kb, across conditions as indicated. The percent expansions for each mutant was divided by the percent expansions for the wild-type CAG-70 strain at the same location. D) Fold over WT percent contractions for CAG-70 repeat, presented as in (C). The red line at 1-fold over WT indicates the same percent instability as wild-type. (^) p≤0.05 compared to CAG-70 wild-type strain in the same condition, (*) p≤0.05 decrease compared to No RFB or Weak RFB (as indicated) in mutant by Fisher’s Exact Test. If no symbol is present, it indicates that the comparisons showed no significant difference. See Tables B and C in S1 Text for exact number of colonies analyzed and percentages for individual assays. See Table K in S1 for P-values.

Based on the increased CAG expansions and contractions observed upon induction of the RFB, we wanted to determine if they were specifically due to RDR. RDR was previously shown to require Rad52 in this system [27–29]. Therefore, Rad52 was deleted to determine the effect on repeat instability. This mutant was tested for both the 1.9 kb and 6.7 kb locations with CAG-70 repeats. In the *rad52Δ* strain, percent expansions decreased in both RFB conditions compared to the *rad52Δ* No RFB condition (Fig 2A), which was significant for the Weak RFB condition for both locations (p = 0.002, p = 0.004) and for Strong RFB at the 6.7 kb location (p = 0.05). This is opposite to the pattern in wild-type cells, where the presence of the RFB increased expansions. Percent expansions also decreased in the Weak RFB and Strong RFB conditions in the *rad52Δ* mutant compared to the levels in the wild-type strain, with the decrease being highly significant in the Weak RFB condition (p = 0.002 at 1.9 kb, p = 0.0004 at 6.7 kb) (Fig 2C). The dramatic decrease in expansions in *rad52Δ* compared to the wild-type strain is evident when comparing the fold changes (starred comparisons, Fig 2C). These data indicate that Rad52, while dispensable for spontaneous expansions, is required for the RFB-dependent CAG repeat expansions observed, implicating RDR in creating the RFB-dependent expansions.

The baseline of contractions in the No RFB condition increased significantly in the *rad52Δ* mutants, 3 to 5-fold over wild-type (Fig 2A and 2D). Unlike for expansions, contractions were not reduced in the *rad52Δ* background upon induction of the RFB but remained high (Fig 2A and 2D). Therefore, Rad52-dependent fork restart is not creating most of the RFB-dependent contractions. However, we did note that the marked RFB-dependent increase in contractions observed in wild-type cells was not as evident in the *rad52Δ* background, especially at the 1.9 kb location (compare the contraction pattern in Fig 2A to Fig 1C), suggesting that some RFB-specific contractions may be suppressed when Rad52-dependent fork restart is not occurring, at least in the Weak RFB situation. Unfortunately, any potential RFB-dependent decrease is hidden by the high baseline level of contractions in *rad52Δ* cells. We conclude that, unlike expansions, RFB-dependent contractions are not fully dependent on Rad52. Therefore, there are also other contraction mechanisms at play that occur independently of the RDR process.

*S*. *pombe* Rad8 (*S*. *cerevisiae* Rad5; *Homo sapiens* HLTF) is involved in the template-switching pathway that could be utilized during fork restart. Since we had previously shown that CAG expansions can occur in a Rad5-dependent manner [40], we wanted to test the role of template switch in the observed RFB-dependent expansions. We first addressed the contribution of Rad8 to RDR using a previously described genetic assay that allows the monitoring of replication slippage occurring during the progression of the restarted fork [29]. Upon induction of the RFB (Strong RFB), the frequency of replication slippage was increased by 18.4-fold and 22.7-fold, compared to the No RFB control, in wild-type and *rad8Δ* cells, respectively (S3A and S3B Fig). This contrasts with the strong reduction previously observed in the absence of Rad52 [29,39]. 2D analysis revealed that the RFB was properly activated in *rad8Δ* cells. We observed a slight increase in the level of arrested forks undergoing Exo1-mediated long-range resection in *rad8Δ* cells compared to wild-type, but the termination signal (*i*.*e*. when the two opposite forks converge within the restriction fragment analyzed) was similar to the wild type level (S2C and S2D Fig). We conclude that Rad8 is dispensable for HR-mediated fork restart. Then, we analyzed RFB-induced CAG repeat instability in the absence of Rad8. Compared to wild-type, *rad8Δ* mutants had a 2-fold increase in expansions in the No RFB condition (p = 0.06) (Fig 2B and 2C). Thus, under normal growth conditions with no *RTS1* sequence, the Rad8 protein appears to protect against repeat expansions, as observed previously in *S*. *cerevisiae* [40,41]. However, unlike in wild-type strains, *rad8Δ* mutants had a decrease in expansions for both the Weak RFB and Strong RFB conditions when compared to the No RFB condition (Fig 2C), although not as strong as the *rad52Δ* phenotype. This suggests that Rad8-dependent template switching causes some of the expansions generated during progression of the restarted fork. For contractions, *rad8Δ* mutants had a significant 3-fold increase compared to wild-type in the No RFB condition (p = 0.0001; Fig 2D), strengthening the conclusion that Rad8 has a protective role at CAG repeats outside of fork restart. Consistent with the expansion data, *rad8Δ* mutants exhibited a significant decrease in contractions for the Weak RFB condition (60% of wild-type levels: 24% contractions in wild-type compared to 14% in *rad8Δ*, p = 0.03), though this wasn’t evident for the Strong RFB condition (Fig 2B and 2D). These data suggest that Rad8 is responsible for some but not all of the RFB-dependent contractions. Since Rad8 is dispensable for RDR to occur, we propose that Rad8-dependent template switch events occur after the fork has restarted by RDR and while it is progressing through the CAG tract. Overall, we conclude that RFB-dependent expansions are occurring during a Rad52- and a Rad8-dependent process. In contrast, the RFB-dependent contractions are only partially dependent on a Rad52- and Rad8-dependent process, and mostly occur by a different mechanism.

### Exo1 and Swi10 dependent mechanisms do not significantly contribute to CAG-70 repeat instability after induction of an RFB

**Fig 3:**
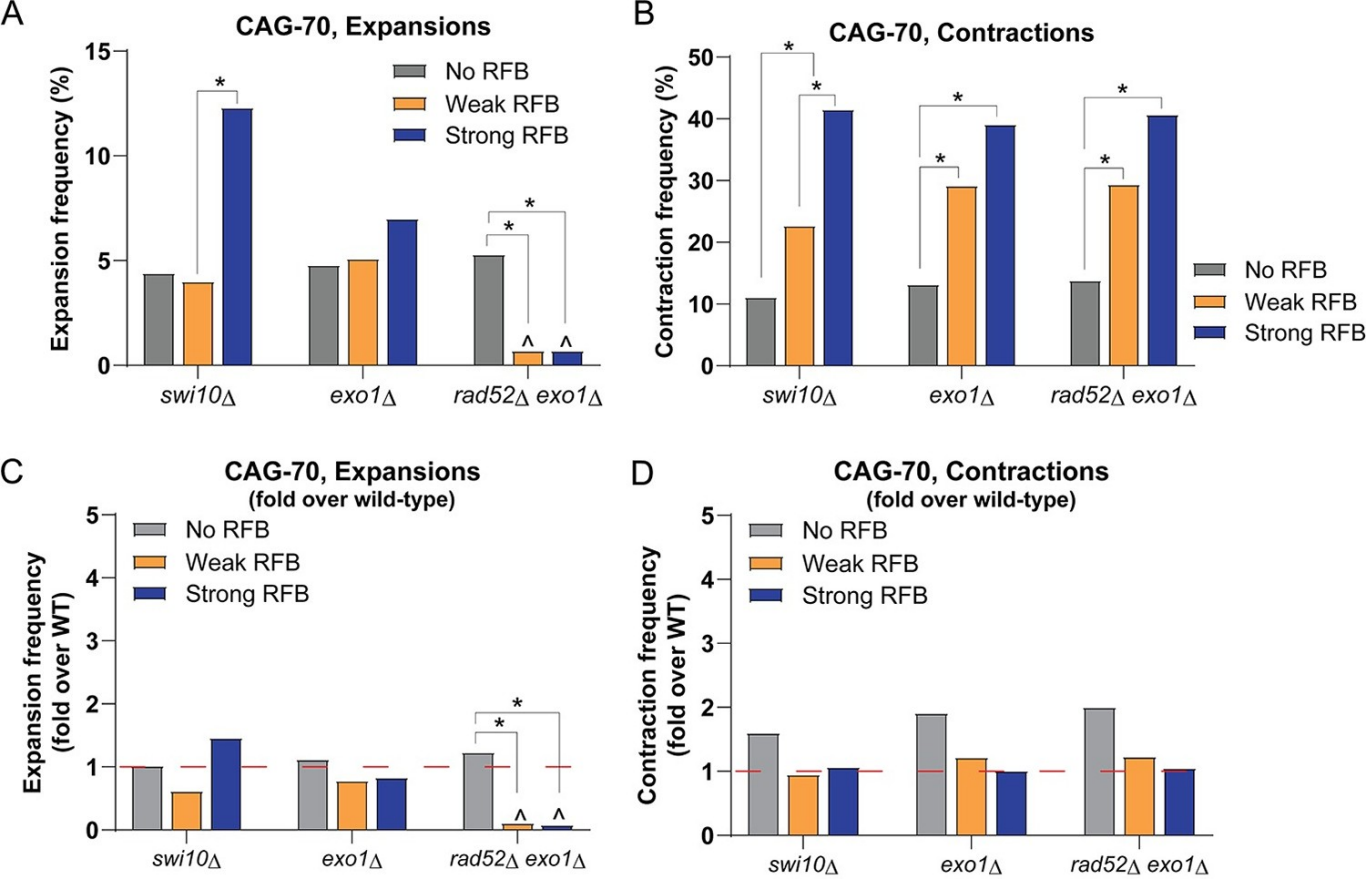
Exo1 and Swi10 mediated mechanisms do not significantly contribute to CAG-70 repeat instability after induction of an RFB. A) Percent expansions for CAG-70 at the 1.9 kb location in *swi10Δ*, *exo1Δ*, and *exo1Δrad52Δ* mutants across the indicated conditions. B) Percent contractions for CAG-70 at the 1.9 kb location in *swi10Δ*, *exo1Δ*, and *exo1Δrad52Δ* mutants across the indicated conditions. C) Fold over WT percent expansions for the CAG-70 repeat at the 1.9 kb location in *swi10Δ*, *exo1Δ*, and *rad52Δexo1Δ* mutants across conditions as indicated, calculated as in Fig 2. D) Fold over WT percent contractions for the CAG-70 repeat, presented as in (C). The red line at 1-fold over WT indicates the same percent instability as wild-type. (*) p≤0.05 compared to No RFB or Weak RFB (as indicated), (^) p≤0.05 compared to CAG-70 in the WT strain in the same condition by Fisher’s Exact Test. If no symbol is present, it indicates that the comparisons showed no significant difference. See Tables D, E, and F in S1 Text for exact number of colonies analyzed and percentages for individual assays. See Table K in S1 Text for P-values.

We observed that RFB-dependent contractions are not fully dependent on Rad52, suggesting they may arise during another process besides Rad52-dependent fork restart. Since long CAG/CTG repeats are known fragile sites that break with some frequency [42] one possibility was that RFB induction caused increased breaks within the CAG tract and subsequent deletions. If this were the case, deletion of Exo1, a 5’ to 3’ exonuclease that processes 5’ ends during DSB repair, would be expected to reduce contractions. Of note, although Exo1 is involved in the long-range resection of nascent strands at forks blocked by the RFB, HR-mediated fork restart occurs in the absence of Exo1 at the same frequency as in wild-type cells [39]. The deletion of Exo1 in the strain containing CAG-70 at the 1.9 kb location did not significantly change the percent expansions or contractions when compared to wild-type in any of the conditions (Fig 3). Similarly, there was no significant RFB-dependent effect in the *exo1Δ* mutant different than that already observed in wild-type cells (Fig 3). Overall Exo1 does not significantly affect RFB-dependent CAG repeat instability. These data argue against DSBs within the CAG tract as being a significant cause of the RFB-dependent contractions observed in wild-type cells. Additionally, since there was no evidence for a suppression of contractions in the *exo1Δ* mutant, most CAG contractions that happen after RFB induction are not due to Exo1-dependent resection at forks traversing or stalled within the CAG tract.

To further rule out an effect of DSB repair in causing RFB-induced contractions, *Sp*Swi10 (*Sc*Rad1-Rad10; *Hs*XPF-ERCC1), a flap endonuclease involved in single-strand annealing (SSA), was tested to determine if the SSA pathway was involved. Deletion of *swi10* in the strain with CAG-70 at the 1.9 kb location did not significantly change the percent expansions or contractions when compared to wild-type (Fig 3). Therefore, Swi10 does not play a role in RFB-independent or RFB-dependent repeat instability, and expansions and contractions are likely not occurring during SSA at DSBs occurring within the CAG tract during fork restart.

The results for *rad52Δ* indicated that CAG contractions in the absence of Rad52 were no longer RFB strength dependent. However, the basal level (No RFB condition) of contractions was significantly increased 3- to 5-fold in *rad52Δ* cells compared to wild-type (Fig 2B and 2D). We were curious how these baseline contractions within the repeat tract in the *rad52Δ* background were generated. Since Exo1 performs resection at DSBs [43] and CAG tracts are known to exhibit increased fragility in the *rad52Δ* background [44], an *exo1Δ rad52Δ* mutant was tested. Indeed, the level of contractions in the No RFB condition decreased from 34% in *rad52Δ* to 13.8% in *exo1Δrad52Δ*, similar to the level of 13.2% in the *exo1Δ* single mutant, indicating that they occurred due to Exo1-mediated resection (Fig 3B and 3D). Thus, these baseline contractions in the *rad52Δ* background could be occurring during spontaneous and stochastic DSBs that occur within the CAG tract in the absence of Rad52, followed by Exo1 processing. When this pathway is eliminated, the remaining contractions in the *exo1Δrad52Δ* mutant were again RFB strength dependent as in wild-type cells (Fig 3B).

As in the *rad52Δ* single mutant, RFB-dependent expansions were almost completely eliminated in Weak RFB and Strong RFB conditions in the *exo1Δrad52Δ* mutant, providing further support that the RFB-induced expansions are Rad52-dependent and thus likely occurring due to HR-mediated fork restart (Fig 3A and 3C).

### Putting the CTG repeat on the lagging strand template creates a strong bias for contractions

**Fig 4:**
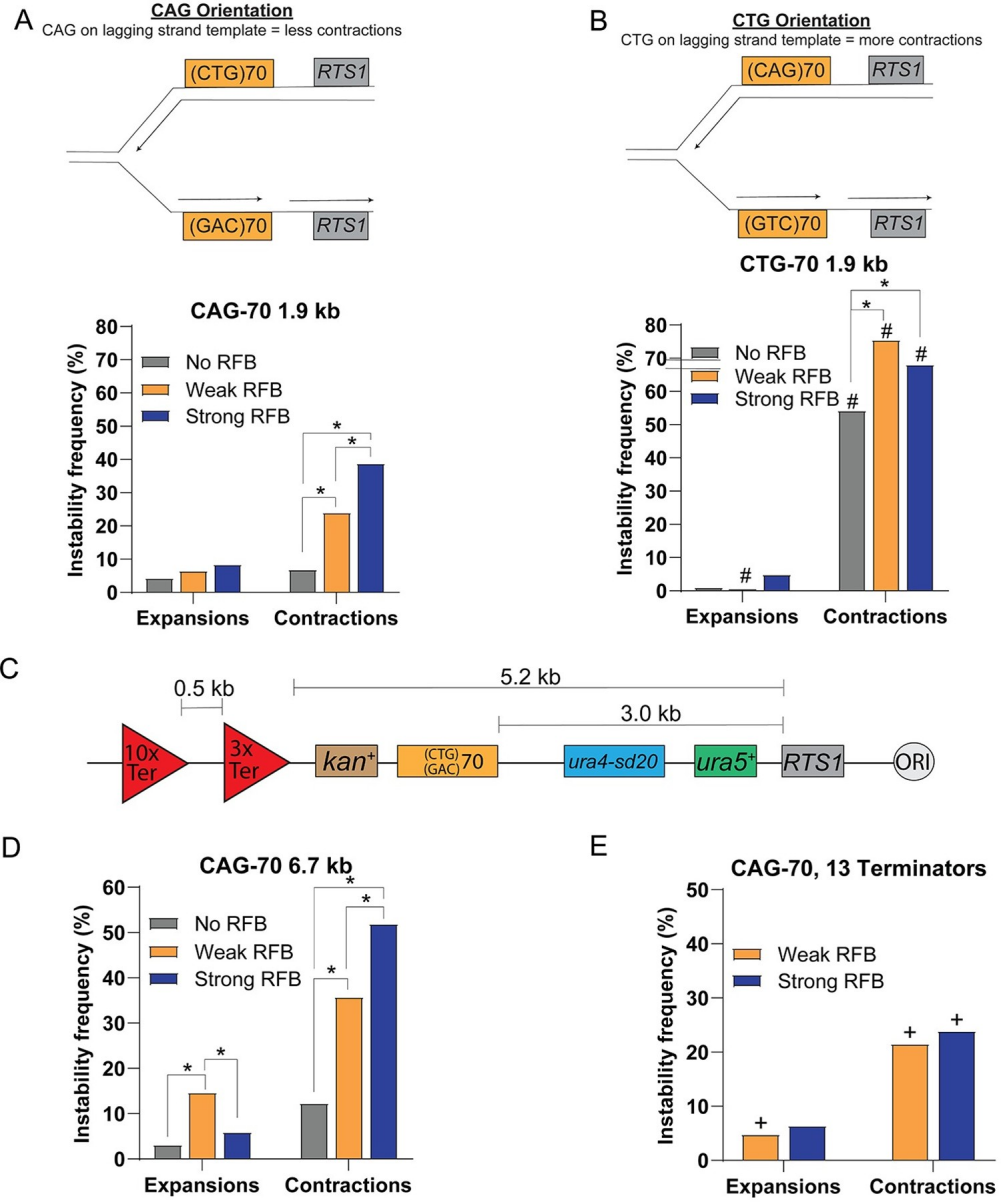
Contraction frequency is influenced by replication direction. A) Schematic of the CAG-70 repeat location 1.9 kb away from the *RTS1* sequence where CAG is on the lagging strand template if replicated by the leftward or restarted fork. CAG-70 WT instability data across conditions as indicated. B) Schematic of the CTG-70 repeat location 1.9 kb away from the *RTS1* sequence where CTG is on the lagging strand template if replicated by the leftward or restarted fork. CTG-70 WT instability data across conditions as indicated. (*) p≤0.05 compared to No RFB or Weak RFB (as indicated), (#) p≤0.05 CTG-70 compared to CAG-70 at 1.9 kb in the same condition by Fisher’s Exact Test. C) Schematic of CAG-70 repeats 3.0 kb away from the *RTS1* sequence. Three Ter2-Ter3 terminators were integrated 5.2 kb from the *RTS1* sequence and an additional ten Ter2-Ter3 terminators 0.5 kb further away to slow replication from the converging rightward fork and increase the probability that the CAG/CTG tract is replicated by the leftward restarted fork. D) CAG-70 WT instability data across conditions indicated. E) CAG-70 instability across conditions indicated in the construct with 13x Ter. (+) p≤0.05 compared to CAG-70 at the 6.7 kb location without terminators in the same condition by Fisher’s Exact Test (suppression of Strong RFB contractions, p = 0.001 to 1.9 kb location without terminators). See Tables A, H, and I in S1 Text for exact number of colonies analyzed and percentages for individual assays. See Table L in S1 Text for P-values.

Since the RFB strength-dependent contractions were not fully dependent on Rad52 this suggested that some occur during a process other than fork restart. The other way an RFB-stalled replication fork can be rescued is by the converging replication fork from the opposite direction. A signal for this converging fork can be seen by 2D gel when the RFB is induced (see S1C Fig ON condition and S3C Fig). This would switch the replication direction of the CAG-70 repeat and put the CTG repeat on the lagging strand template, an orientation known to be more prone to contractions in *S*. *cerevisiae* [33–35]. To determine if such a change in fork direction would have a similar effect on the CAG repeat integrated into this genomic location in *S*. *pombe*, the repeat was flipped to create the CTG-70 orientation (CTG on lagging strand template) at the same 1.9 kb distance from *RTS1* (Fig 4A and 4B). Significantly more contractions were observed in the CTG-70 strain when compared to the CAG-70 strain in this orientation, reaching 54% (p = 0.0001 to CAG-70) and confirming that CTG on the lagging strand template is highly contraction prone at this chromosomal locus in fission yeast (Fig 4A and 4B). Both the Weak RFB and Strong RFB conditions exhibited an even greater frequency of contractions, 75% and 68%, respectively. Interestingly, the expansion frequencies were opposite to the CAG orientation pattern, with fewer expansions in the Weak RFB (0.7%) compared to the Strong RFB condition (4.9%). This can be explained by the strong RFB now causing the CTG-70 tract to sometimes be replicated by the converging fork such that CAG is now again on the lagging strand template and less likely to contract, and the hairpin-forming CTG repeat is now on the nascent lagging strand, a situation known to lead to expansions (see [1] for review) (Fig 4). These data are consistent with the idea that the RFB-dependent contractions in the CAG-70 strain were largely due to a change in fork direction, which happens more frequently when there is a strong RFB.

### Replication by the converging replication fork contributes to CAG-70 contractions

To further test the idea that some RFB-dependent contractions were due to a switch in replication direction such that the CAG/CTG tract was replicated by the converging (rightward) fork more frequently, a CAG-70 tract was integrated 3.0 kb downstream of the *RTS1* site in a strain that had 13 copies of the Ter2-Ter3 rDNA replication fork barrier, which delays the converging replication fork (Fig 4C) [32,45]. In this strain 80% of forks blocked by the RFB are restarted by RDR [32]. Indeed, when the converging replication fork is delayed, contractions were significantly reduced in the Strong RFB condition when compared to constructs without the Ter sequences, from 39% (1.9 kb location) or 52% (6.7 kb location) to 24%, (p = 0.001 compared to CAG at 1.9 kb or p = 0.0001 compared to CAG at 6.7 kb) (Fig 4D and 4E). Altogether, these data support the conclusion that a large proportion (at least half) of the RFB-dependent contractions are due to a switch in replication direction due to a failure of fork restart at the RFB, resulting in the hairpin-forming CTG repeat being on the lagging strand template.

There was no change in expansion frequencies compared to strains without Ter (Fig 4D and 4E), indicating that expansions are not created by the converging rightward fork and consistent with them occurring due to replication by the restarted (leftward) fork.

### Early steps of fork processing and restart do not make a significant contribution to CAG-70 repeat instability during fork restart

**Fig 5:**
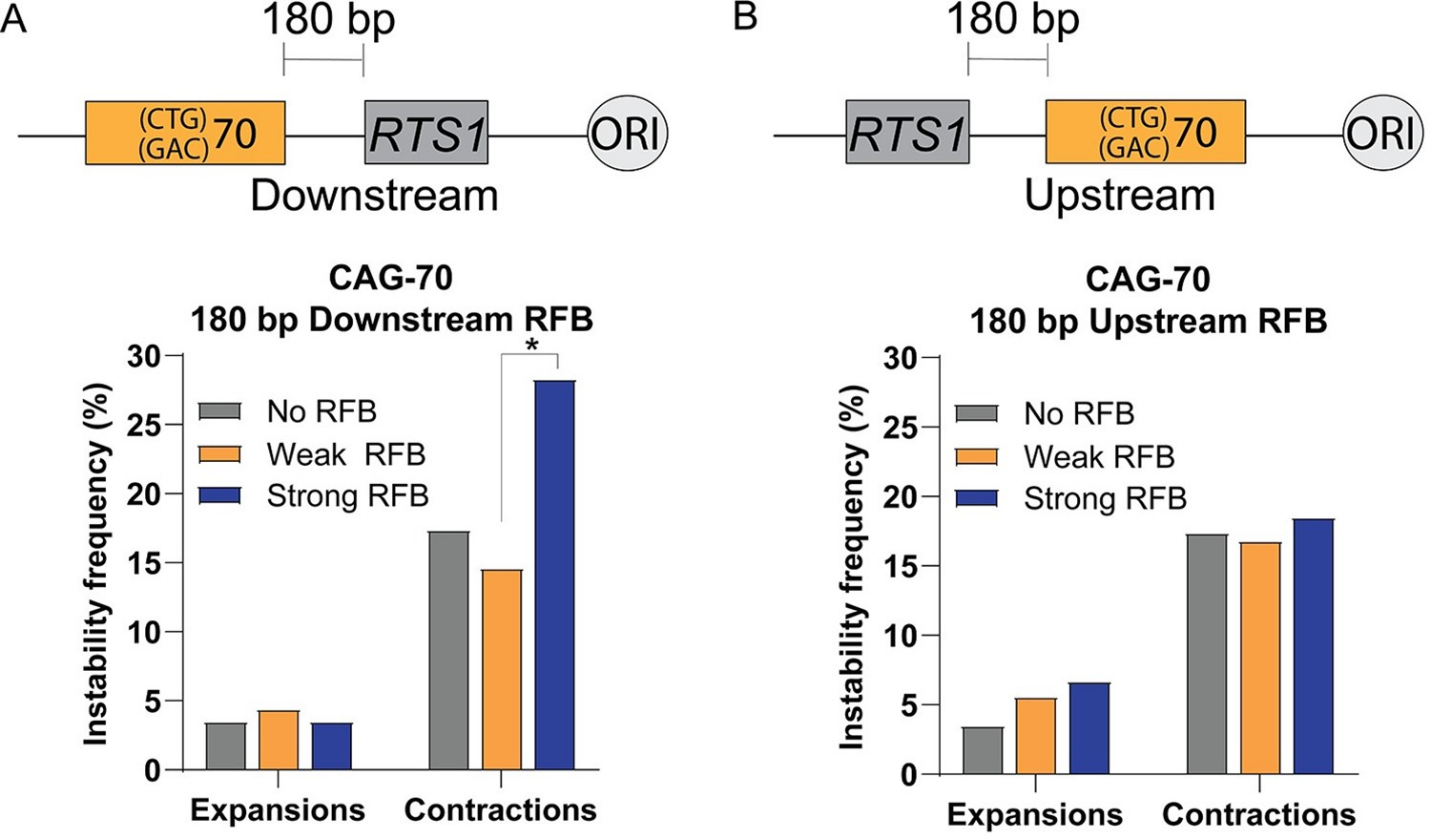
Migrating D-loops and reversed forks are not a significant source of CAG-70 repeat instability during fork restart upon induction of an RFB. A) Schematic of CAG-70 repeats 180 bp downstream from the *RTS1* sequence. CAG-70 180 bp Downstream RFB instability data across conditions as indicated. B) Schematic of CAG-70 repeats 180 bp upstream from the *RTS1* sequence. CAG-70 180 bp Upstream RFB instability data across conditions as indicated. (*) p≤0.05 compared to Weak RFB by Fisher’s Exact Test. See Table J in S1 Text for exact number of colonies analyzed and percentages for individual assays. See Table L in S1 Text for P-values.

In order to test if the early steps of fork-processing necessary for HR-mediated fork restart contribute to repeat instability, the CAG repeats were integrated just upstream or downstream of the RFB (Fig 5). When inserted 180 bp downstream from the RFB, the early-stage restarting fork, which may be in the form of a D-loop and could be somewhat different than the established restarted fork, would encounter the CAG tract and need to traverse through it. At this location all three conditions (No RFB, Weak RFB and Strong RFB) had a similarly low level of expansions (3.5–4.4%; Fig 5A). The RFB-specific increase in expansions observed at the locations further downstream was not observed, and for the Weak RFB condition there were only 1/3^rd^ as many expansions as at the 6.7 kb location (Fig 5A). Surprisingly, this indicates that replication structures close to the fork restart position, such as migrating D-loops, do not significantly contribute to the RFB-dependent repeat expansions observed at the CAG locations further downstream (2–7 kb) from the RFB. The error-prone mechanism replicating the repeats farther from the RFB (e.g. the δ/δ replication fork) might not be established so close to the RFB.

For contractions at the 180 bp downstream location, there was a marked increase for the Strong RFB condition when compared to No RFB (p = 0.06) or Weak RFB (p = 0.01). However, the Weak RFB did not have an increase in contractions compared to No RFB as was observed at the other locations (Fig 5A). When compared to CAG-70 at the 1.9 kb location, there was a significant decrease in percent contractions for both Weak RFB and Strong RFB conditions (0.6 and 0.7-fold, respectively). This data is consistent with the model that most contractions occur due to the CAG/CTG tract being replicated by the converging fork when there is a replication barrier and suggests that this happens less frequently when the tract is so close to the RFB, especially if the RFB is weak. We did note a significant increase in contractions for the No RFB condition (2.5-fold increase compared to the 1.9 kb location) (Fig 5A). Therefore, the CAG repeats are generally more unstable at this location, suggesting a role for the specific genomic context in altering instability. Altogether, though the basal level of contractions were increased at the 180 bp downstream location, the RFB-dependent increase in instability was either absent or much reduced compared to the 1.9 and 6.7 kb locations.

The CAG-70 repeats were also integrated 180 bp upstream from the RFB to understand if fork reversal or fork resection, which could help restart the replication fork, provides an opportunity for generating repeat instability (Fig 5B). At this location, any reversal or processing of the stalled fork induced by the RFB would occur within the CAG tract. Surprisingly, there was no RFB-dependent difference in percent expansions or contractions across all conditions (Fig 5B). Therefore, we did not find any evidence for RFB-induced fork reversal or processing within the repeat as a significant source of repeat instability.

### Msh2 is required for CAG expansions in *S*. *pombe*, showing a conserved mechanism for MMR proteins in generating expansions

**Fig 6:**
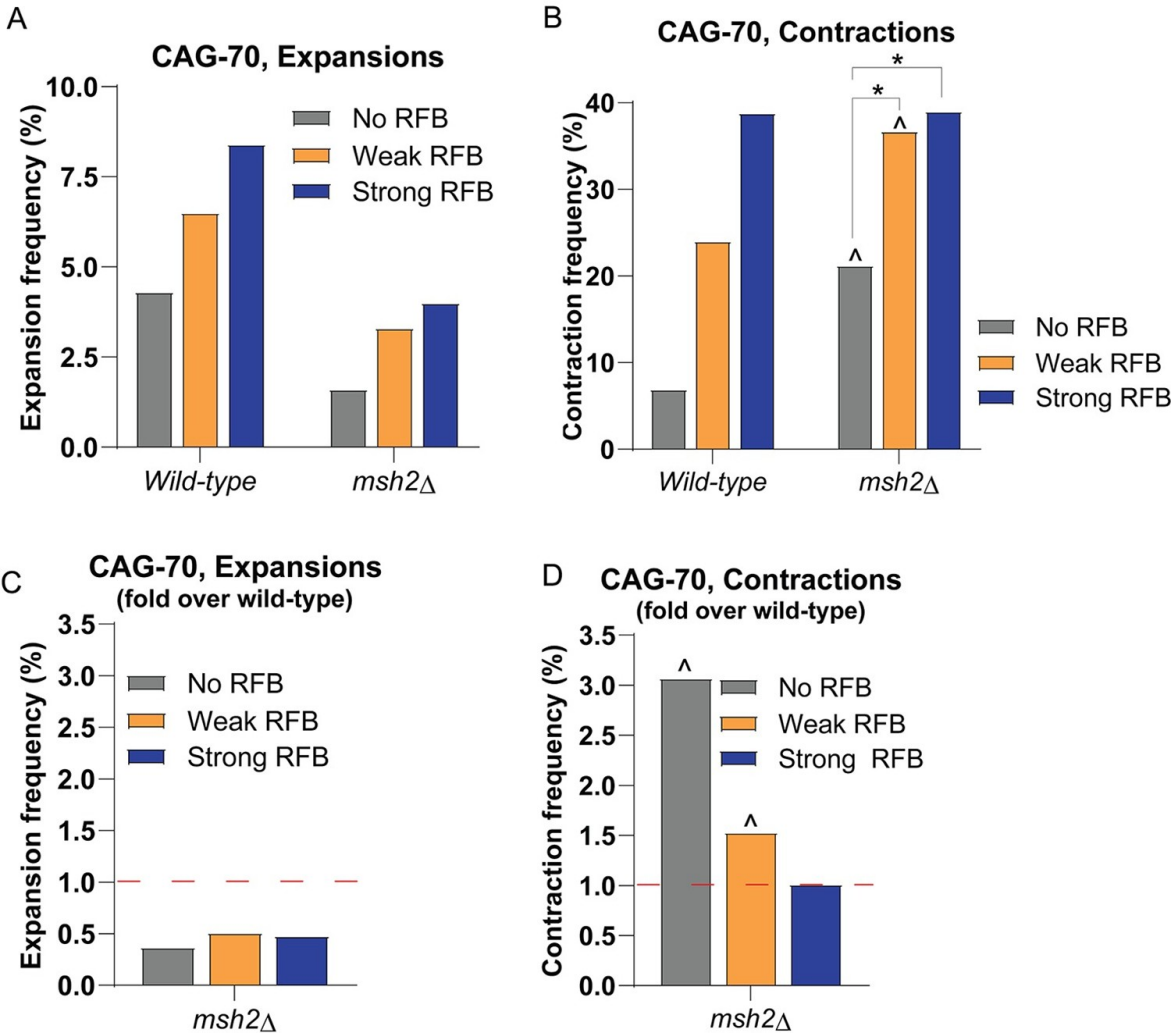
Msh2 is required for CAG-70 repeat expansions independent of replication fork restart. A) Percent expansions for CAG-70 at the 1.9 kb location in a *msh2Δ* mutant across the indicated conditions. B) Percent contractions for CAG-70 at the 1.9 kb location in an *msh2Δ* mutant across the indicated conditions. C) Fold over WT percent expansions for CAG-70 repeat in an *msh2Δ* mutant at 1.9 kb across conditions as indicated. The percent expansions for each mutant was divided by the percent expansions for the wild-type CAG-70 strain at the same location. D) Fold over WT percent contractions for CAG-70 repeat, presented as in (C). The red line at 1-fold over WT indicates the same percent instability as wild-type. (*) p≤0.05 compared to No RFB or Weak RFB (as indicated), (^) p≤0.05 compared to CAG-70 at 1.9 kb in the WT strain in the same condition by Fisher’s Exact Test. See Table G in S1 Text for exact number of colonies analyzed and percentages for individual assays. See Table K in S1 Text for P-values.

In mouse models for CAG repeat expansion disorders, the MutSβ complex Msh2-Msh3 is responsible for the majority of expansions in non-dividing somatic cells and also some repeat instability during intergenerational transmission, with expansions severely reduced in the absence of the MSH2 gene (see [1,46] for review). Although it has been established that the Msh2-dependent expansions in non-dividing cells mostly occur during gap repair processes such as base excision repair (BER), it is unclear whether Msh2-Msh3 also plays a role in replication-dependent expansions. Using 2D gel analysis, it was shown that fork stalling by an expanded (CAG/CTG)98 tract in *S*. *cerevisiae* was dependent on Msh2 [11]. Therefore, we tested whether Msh2 plays a role in the RFB-dependent expansions within the CAG tract. Deletion of Msh2 in the strain with CAG-70 at the 1.9 kb location led to an overall decrease in percent expansions for all conditions when compared to wild-type: from 4.3–8.4% in wild-type to 1.6–4% in *msh2Δ*, an average decrease of 2.3-fold (Fig 6). This indicates that expansions were suppressed in the *msh2Δ* strains independent of RFB status. In contrast, baseline contractions significantly increased in the *msh2Δ* mutant (3-fold increase over wild-type for No RFB) (Fig 6). However, the further increase in contractions due to the RFB was not altered by the absence of Msh2, implying no role for Msh2 in causing RFB-dependent contractions. In summary, the *msh2Δ* mutant showed a decrease in expansions compared to the wild-type condition but an increase in contractions. These data indicate that the presence of Msh2 promotes expansions, showing a conserved mechanism for MMR proteins in generating expansions in *S*. *pombe*, similar to its role in higher eukaryotes. Since this phenotype is RFB-independent, it indicates that Msh2-dependent expansions are not occurring during replication fork restart, but rather during another process such as gap repair.

## Discussion

By using a strong site-specific and inducible RFB placed next to an expanded CAG tract, we were able to separate out the effects of the natural fork stall created by the CAG tract (which would still occur in the no RFB condition) and directly measure the effects of RDR on CAG repeat instability. This allowed us to pinpoint that the most expansion-prone condition is a fully restarted replication fork progressing through the repeat tract, suggesting that the properties of the restarted fork lead to increased hairpin formation and strand slippage on the nascent strands. The most expansion-prone condition occurred when the CTG sequence was on the lagging nascent strand. The most contraction-prone situation was when the strong fork barrier led to the repeat being replicated such that the CTG sequence was on the lagging strand template. This orientation dependence of repeat instability is similar to what has been observed previously in *S*. *cerevisiae* [33–35] and human cells [36–37,47].

**Fig 7:**
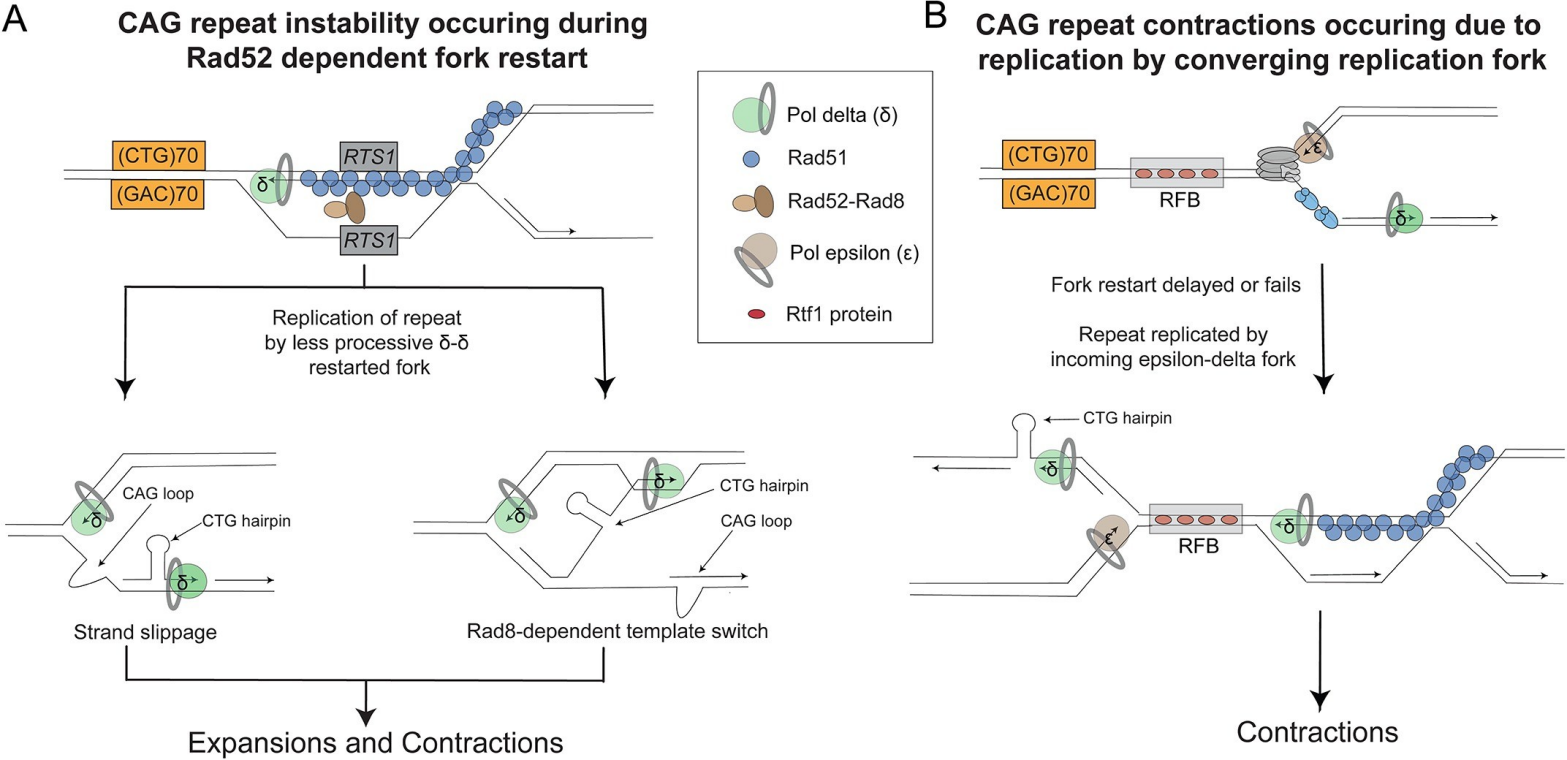
Model for generation of CAG/CTG expansions and contractions during recovery from a replication fork barrier. (A) The restarted δ–δ fork is highly prone to slippage and *Sp*Rad8/*Sc*Rad5/*Hs*HTLF-dependent template switching to generate both repeat expansions and contractions. For illustrative purposes, structures are shown to form on the lagging strand, which has been shown to contain extensive single-strandedness during replication of the uncoupled δ–δ fork [45]. (B) A strong fork barrier causes the repeat tract to be replicated in the opposite direction by the rightward converging fork, altering the instability profile. For the orientation studied here, that results in CTG repeat placement on the lagging template strand, where hairpin formation will lead to contractions.

The condition of putting the CAG tract only 180 bp in front of the CAG tract caused a situation where the initial restart event, presumably a D-loop type structure, would be occurring within the CAG repeat. We expected that this event would be particularly error-prone and susceptible to slippage or misalignments within the repeat tract, causing the highest level of instability. Surprisingly, this was not the case. Instead, the CAG tracts further away from the stall (2–7 kb away) showed a more pronounced RFB-dependent instability, which was more dramatic at the 6.7 kb location compared to the 1.9 kb location. For the CAG contractions, this can be largely attributed to the fact that the further the repeat is from the RFB, the more likely it is to be replicated by the converging fork while the closer fork that would normally reach the repeat tract first is stuck at the barrier. However, this phenomenon did not explain the increase in expansions, which also increased as the repeat was further from the barrier, especially for the weak barrier situation where the converging fork is unlikely to replicate the repeat. These RFB-dependent expansions are best explained by being replicated by a restarted fork. Supporting this conclusion, these expansions were almost completely dependent on Rad52, suggesting that they were due to the restarted fork which is dependent on HR-mediated RDR. It has previously been shown by the Carr lab that the RDR occurring after this same RFB proceeds by semi-conservative replication but with Polδ replicating both leading and lagging strands within the distance we tested [32]. The Whitby lab reported that the liability of a restarted fork to collapse and template switch, especially when replicating repeated sequences, occurs up to 75 kb away from the restart site [31,48]. Therefore, we propose that this δ-δ replication fork is inherently less processive and more prone to slippage or dissociation and re-association, leading to an increase in CAG instability (Fig 7A). The partial dependence of expansions on *Sp*Rad8^*Sc*Rad5/*Hs*HLTF^, which is dispensable for fork-restart, suggests that Rad8 mediates increased template switching of the restarted fork, leading to repeat expansions. Our data suggests that the longer the δ-δ fork progresses the greater the chance that a slippage or template switch occurs to cause a repeat length change.

Interestingly, the large increase in contractions in the RFB On/Strong RFB condition (40–50%) compared to the RFB Off/Weak RFB condition (25–35%) could be largely attributed to a replication direction switch due to the CAG/CTG repeat being replicated by the converging (rightward) fork (Fig 7B). This was most evident from the experiment where terminators slowed the converging fork. In this case, the further increase in CAG contractions normally observed in the Strong RFB condition was completely eliminated (Fig 4). However, there were still ~20% contractions remaining, the same as for the Weak RFB condition but more than the No RFB control (~10%), suggesting that these contractions occurred during the fork restart process. Therefore, contractions occurred by two different mechanisms: (1) during fork restart (Fig 7A), and (2) due to a switch in replication direction that placed the CTG repeat on the lagging strand template (Fig 7B). There appeared to be a bias towards small contractions in the strong RFB condition at 1.9 kb and in both strong and weak RFB conditions at 6.7 kb, suggesting that contractions generated by replicating the CTG strand on the lagging strand template (something that occurs more often in these conditions) tend to be smaller. Another interesting observation is that some Strong RFB-specific contractions occurred even at the CAG tract location 180 bp downstream of the *RTS1* sequence, suggesting that some forks stalled by the RFB never restart and even sequences right after the RFB are replicated by the converging fork. Effects of changing replication fork direction on repeat instability have been noted before in multiple model systems, from yeast to human cell lines (reviewed in [1]). Indeed, mapping of replication origins near expandable CAG repeats in human cells indicates that replication of the HD and DM1 loci (where expansions can cause Huntington’s disease, or myotonic dystrophy type 1, respectively) puts CAG on the lagging strand template and CTG on the nascent lagging strand, consistent with the observed predisposition for expansions at these loci [49,50].

Locating the CAG tract just behind the RFB in the region shown to be resected during fork stalling at the RFB [39], might have been expected to increase instability. For example, template strands exposed by resection could fold into hairpin structures to cause contractions, or hairpins formed at the exposed end of a resected reversed fork could lead to expansions [16]. However, our data did not show an increased level of RFB-dependent instability at this location, demonstrating that resection was not the main driver of RFB-dependent repeat instability. Rather, the most consistent trend was an increased basal level of contractions at the two locations close to the origin (20% compared to 7% when the CAG tract was further away).

Note that our study does not address whether expansions occur during a BIR process initiated at a broken fork, because the barrier system we used does not cause a detectable level of breaks in wild-type cells. Rather, restart in this RFB system is dependent on single stranded gap formation and Rad52-dependent strand invasion [29]. At the natural stall caused by a long CAG tract, there could be spontaneous breaks that lead to a broken fork initiating the restart process in a BIR-like process termed broken fork repair (BFR). This was a proposed mechanism mediating CAG expansions in a *mre11*Δ background that showed increased breaks and Rad52-dependent expansions [44], and in a system measuring large-scale CAG expansions that showed dependence on both Rad52 and Pol32, which are required for BIR [51]. Recently, evidence for POLD3-mediated BIR initiated at CAG repeats in human cells was obtained [52]. Therefore, we are not excluding that this is also a mechanism for creating replication-dependent CAG repeat expansions. This or other mechanisms could be occurring in the No RFB condition to produce the basal level of 3–4% expansions observed, which is still a very high frequency compared to other types of mutational events. What our results do indicate is that if a restarted fork is initiated at the beginning of a long repeat tract, with or without a break, that subsequent Polδ–Polδ replication through the remainder of the tract will be prone to generating repeat expansions. It was also previously shown that there is an increased frequency of repeat-induced mutations (RIM) up to 10 kb from several structure-forming repeats, including GAA, CGG, inverted repeats, and internal telomeric repeats [53]. Depending on the system, RIM was either partially or fully dependent on Polζ and hypothesized to occur during either BIR or DSB repair occurring after breakage of the repeat [53]. Since we were not able to detect a role for either Exo1-mediated resection or Swi10-mediated single strand annealing, the RFB-mediated repeat instability we observed is likely different from DSB-induced RIM.

What is the potential mechanism by which expansions and contractions occur during Polδ–Polδ RDR synthesis? We were able to exclude Swi10-dependent events, suggesting they are not due to breaks followed by SSA or MMEJ. Similarly, since deletion of Exo1 had no effect, they are not likely due to repair of HR-mediated breaks or resection of single-stranded gaps. It is possible that 5’ flap processing of the lagging strand is disrupted, an event known to lead to CAG/CTG expansions [42,54]. However, this process also occurs during normal replication and is not likely to be altered at the δ–δ fork. One likely explanation is that Polδ-mediated replication of both the leading and lagging strands is less coordinated or less processive, leading to more opportunities for dissociation of the nascent strands, hairpin formation, and re-alignment out of register to cause a repeat length change. Consistent with this idea, a recent report from the Carr lab shows that leading and lagging strand synthesis are uncoupled during progression of the restarted fork, leading to an increase in single-stranded DNA and suggesting that the lagging strand template remains as a gap that is filled in by Pol δ later [45]. Extensive single-stranded DNA on the gapped lagging strand template would provide ample opportunity for DNA structure formation and slippage during fill-in by Pol δ (Fig 7A). Intriguingly, elimination of the template switch pathway by deleting *Sp*Rad8^*Sc*Rad5/*Hs*HLTF^ reduced RFB-dependent CAG instability. This result predicts that there is increased template-switching at the δ–δ fork and that many expansions and contractions are happening during an aberrant template switching process (Fig 7A). Further investigation of the special properties of the δ–δ fork will be necessary to determine a more precise cause of repeat instability.

Altogether, our data suggest that replication restart, though generally protective, does come at the cost of a more error-prone fork, and that this may be especially deleterious during replication of repetitive DNA regions. Our results implicate replication fork restart as a mechanism that could lead to disease-causing repeat expansions in dividing cells. Potentially relevant to our findings, replication progression through an expanded CTG repeat at the DM1 locus is reduced compared to non-expanded controls, with altered fork progression toward the repeat [50]. Several of the expandable CAG loci in human cells, including the DM1 locus, are flanked by binding sites for the CTCF chromatin insulator protein. Cleary et al. showed that placing a CTCF binding site between an origin and a CAG repeat reduced the replication efficiency of a SV40 plasmid, suggesting that CTCF could be responsible for some of the fork slowing observed in cells [50]. If CTCF or other proteins bound near a repeat act as replication fork barriers, that would increase the chance of a restarted fork replicating the repeat tract. More generally, tolerance to replication stress by fork restart is an important pathway in both normal and cancer cells, but our data reveal that this pathway comes at the cost of a high chance of instability of repetitive sequences replicated by this mechanism.

## Materials and methods

### Yeast strains and strain construction

Standard procedures were used for *S*. *pombe* cell growth and medium preparation [55]. Lab strains are listed in Table N in S1 Text. The original replication fork barrier system strains were constructed in the S. Lambert lab [29]. The RFB is ~ 5 kb from ori 3004/3005 on *S*. *pombe* chromosome 3. The CAG/CTG repeat was inserted either 1.9 kb or 6.7 kb from the *RTS1* sequence by homologous recombination. To do this, a (CAG/CTG)70 tract was cloned into a pgEM-5ZF(-) plasmid vector that contained the KanMX6 selectable marker flanked by 434 bp and 478 bp of sequence homology to the 3’UTR of the *mug135* gene. The Spe1-Sph1 restriction fragment was transformed into YC13 (containing the *RTS1*-RFB) and YC6 (without the *RTS1*-RFB) to integrate repeats 6.7 Kb away from the RFB. To integrate the repeats at 1.9 Kb from the RFB, a PCR fragment containing the repeat and the KanMX6 marker was amplified from the same plasmid using primers containing 100 nt sequence homology to the 3’UTR of *ura4* gene (KF124 and KF125) and transformed in YC6 and YC13. To integrate the repeats in the strain containing the Ter barriers, a PCR fragment containing the repeat and the KanMX6 marker was amplified from the same plasmid using primers containing 100 nt sequence homology to the downstream region of the 3’UTR of the *ura4* gene (KF130 and KF131) and transformed in YC266 [39]. Primer sequences are listed in Table O in S1 Text. In CAG-70 strains, 70 CAG repeats were on the lagging strand template if replicated by ori 3005/3006; CTG-70 indicates the opposite orientation. Strains with the *RTS1* were maintained on EMM-glutamate media and the *RTS1* was kept inactive (Weak RFB) by adding 60 uM thiamine to the media.

### Replication fork barrier fork restart assay for CAG repeat instability

Cells were plated on YE plates for single colonies. The CAG or CTG repeat tract was amplified from yeast colonies using primers (CTGrev2/T720-B) (Table O in S1 Text) spanning the repeats to confirm correct tract length. Colonies with the correct tract length were patched onto both EMM glutamate +thiamine (Rtf1 repressed) and EMM glutamate -thiamine (Rtf1 expressed) plates and grown at 30°C for about 72 hours. Half a colony was inoculated into 3mL of the corresponding type of liquid media and grown at 30°C shaking for 22 hours to OD_600_ 1–2. 50–150 μL liquid culture was inoculated into 5 ml of the corresponding type of liquid media and again grown at 30°C shaking for 24–36 hours to an OD_600_ of 1–2. Cells were plated onto YE plates and grown for 3–5 days at 30°C. PCR analysis was done on single colonies using primers (CTGrev2/T720-B, Table O in S1 Text) spanning the repeats to assess tract length. High resolution 2% metaphor agarose gels were used to size the PCR products. Expansions and contractions were determined by reference to a DNA ladder. Using ImageJ, a line was drawn at the starting repeat size and using ladder bands located in multiple lanes across the gel as a reference point. PCR products running above the drawn line were scored as expansions, and below the line as contractions (S2C Fig). Lanes with more than one PCR product were scored as an expansion or contraction if at least 50% of the signal intensity was contained in the band with an altered size. For the CAG repeat sizing analysis, distances were measured between the middle of the wells in the gel and the middle of the bands. First, a standard curve was created using a 100 bp ladder (Thomas scientific) to determine the correlation between base pairs and distance from the well. Then, each CAG repeat PCR product was measured and the number of base pairs in the PCR product was calculated using the standard curve. Lastly, the CAG repeat size was binned according to repeat length. The unaltered (CAG)70 PCR fragment size (repeat + amplified flanking sequence) was 343 bp and each bin was 10 repeats (30 bp). Expansions were considered above 75 repeats and contractions below 65 repeats. A two-sample Kolmogorov-Smirnov (KS) test was used to determine if there were significant differences in the repeat length distributions between conditions. An Advanced Analytical fragment analyzer was also used to size some CAG repeats by capillary electrophoresis in order to confirm suspected expansions.

### Replication slippage assay

The frequency of Ura^+^ revertants using *ura4-sd20* allele was performed as follows. 5-FOA resistant colonies were grown on plates containing uracil and thiamine for 2 days at 30°C and subsequently inoculated into EMM glutamate (EMMg) supplemented with uracil for 24 h. Then cultures were diluted and plated on EMMg complete (for cell survival) and on EMMg without uracil both supplemented with 60 μM thiamine. After 5–7 days incubation at 30°C colonies were counted to determine the frequency of Ura^+^ revertants.

### 2DGE analysis of replication intermediates

Analysis of replication intermediates by two-dimensional gel electrophoresis (2DGE) was performed as described in [56]. Briefly, exponentially growing cells (2.5 × 10^9^ cells) were treated with 0.1% sodium azide and subsequently mixed with frozen EDTA (of final concentration at 80 mM). Genomic DNA was crosslinked with trimethyl psoralen (0.01 mg/mL) added to cell suspensions for 5 min in the dark. Next, cells were irradiated with UV-A (365 nm) for 90 s at a constant flow of 50 mM/cm^2^. Subsequently, cell lysis was performed by adding lysing enzymes at a concentration 0.625 mg/mL and zymolyase 100T at 0.5 mg/mL. The spheroplasts thus obtained were embedded in 1% low melting agarose plugs and incubated overnight at 55°C in a digestion buffer with 1 mg/mL of proteinase K. Plugs were then washed with TE buffer (50mM Tris, 10mM EDTA) and stored at 4°C. Digestion of DNA was performed using 60 units per plug of restriction enzyme AseI or EcoRV, and samples were treated with RNase and beta-agarase (NEB, M0392L). Melted plugs were equilibrated to 0.3M NaCl concentration. Replication intermediates were purified using BND cellulose (Sigma, B6385) poured into columns. RIs were enriched in the presence of 1M NaCl 1.8% caffeine (Sigma, C-8960), precipitated with glycogen and migrated in 0.35% agarose gel (1xTBE) for the first dimension. The second dimension was run in 0.9% agarose gel (1xTBE) supplemented with EtBr. Next, DNA was transferred to a nylon membrane in 10x SSC. Finally, membranes were incubated with ^32^P radiolabeled ura4 probe in Ultra-Hyb buffer at 42°C. The signal from replication intermediates was collected with phosphor-imager software (Typhoon-trio) and quantified by densitometric analysis with ImageQuantTL software (GE healthcare).

## Supporting information

S1 Fig Analysis of fork stalling at CAG/CTG repeats and the RTS1-RFB. (A) Diagram of restriction fragments and probes used within the construct containing the CAG/CTG repeats integrated 6.7 kb downstream the *RTS1*-RFB. (B) examples of 2D gel analysis within the EcoRV restriction fragment containing the CAG-70 repeat tract in indicated strains and conditions (Off: Rtf1 is repressed, On: Rtf1 is expressed). Red brackets indicate the location of the CAG-70 repeats within the ascending arc. (C) Examples of 2D gel analysis within the AseI restriction fragment containing the RTS1-RFB in indicated strains and conditions (Off: Rtf1 is repressed, On: Rtf1 is expressed). Blue arrows indicate fork stalling at the *RTS1*-RFB. Weak fork stalling was detected when Rtf1 is repressed (referred to as Weak RFB condition) compared to the strain devoid of *RTS1* sequence (top panels). A stronger stall (white arrows) was observed upon Rtf1 expression (Strong RFB condition). A signal for the converging fork can be seen coming off the top of the Y arc when the RFB is induced (see S1C Fig ON condition, hook-shaped signal, diagrammed in S3C Fig)
(TIF)

S2 Fig Sizing Analysis of Expansions and Contractions. Analysis depicting CAG repeat contraction and expansion sizes for wild-type strains with a (CAG)70 starting tract size at the A) 1.9 kb and B) 6.7 kb locations across indicated conditions. Sizes were grouped in bins of 10 repeats (30 bp). Expansions were considered above 75 repeats and contractions below 65 repeats. A two sample KS statistical test showed significant differences in the repeat length distributions between conditions No vs. Weak RFB, No vs. Strong RFB, and Weak vs. Strong RFB for the 1.9 kb location; and No vs. Weak RFB and No vs. Strong RFB for 6.7 kb location (p < 0.005 for all. See Table M in S1 Text for a listing of the number of times each length was observed and p-values. (C) Four representative gels that were used to analyze the size of PCR products and determine their approximate size are shown. Bands above the drawn line were scored as expansions (E), and bands below the line as contractions (C). Lanes with more than one PCR product (intact and changed) were scored as an expansion or contraction if at least 50% of the signal intensity was contained in the band with an altered size.
(TIF)

S3 Fig Rad8 is dispensable for RDR. (A) Diagram of constructs containing the reporter allele *ura4-sd20* (red bars) associated with the RFB (*t-ura4sd20<ori*) or not *(t-ura4sd20-ori*). The non-functional *ura4-sd20* allele contains a 20-nt duplication flanked by micro-homology and is located downstream of the RFB. Upon activation of the RFB, ura4-sd20 is replicated by the restarted fork liable to replication slippage, resulting in the deletion of the duplication and restoring a functional *ura4*^*+*^ gene to generate Ura+ cells. As a control, the construct lacking the *RTS1* sequence was used to monitor the spontaneous frequency of replication slippage with no RFB present. (B) Frequency of Ura^+^ cells in indicated strains. Values are means from n independent biological samples and error bars indicate standard deviation. (C) Top panel: Scheme of replication intermediates (RI) analyzed by neutral-neutral 2DGE of the AseI restriction fragment in RFB ON conditions, as described on S1 Fig. Signals corresponding to converging fork, arrested fork and resected fork (tail signal) are indicated [27,39]. Bottom panels: Representative RI analysis in indicated strains. The ura4 gene was used as probe. Numbers indicate the percentage of forks blocked by the RFB ± standard deviation. (D) Quantification of resected fork and converging fork in indicated strains. Values are means from n independent biological samples and error bars indicate standard deviation. p values were calculated using the non-parametric Mann Whitney test.
(TIF)

S4 Fig Comparison of (CAG)70 instability at different chromosomal locations. (A) Summary of frequency of CAG-70 expansions detected at the indicated locations. (B) Summary of frequency of CAG-70 contractions detected at the indicated locations. The CAG repeat is at the same location, replacing the *RTS1* sequence (~ 5 kb from ori 3004/3005) in the No RFB 180 bp before and after locations, thus the instability data is the same for those two bars.
(TIF)

S1 Text Supplementary Tables. The S1 text contains Tables A through O. Tables A to J contain raw CAG instability data for all the strains tested. Tables K and L contain p values for instability comparisons between strains. Table M contains the CAG expansion and contraction size distributions for the wild-type strain. Table N contains the *S*. *pombe* strains used in this study. Table O contains the primers used in this study.
(DOCX)
