# Supplementary figures and images for: Restarted replication forks are error-prone and cause CAG repeat expansions and contractions
Source: PLoS Genet. 2021 Oct 21;17(10):e1009863. doi: 10.1371/journal.pgen.1009863 (PMC8562783; doi:10.1371/journal.pgen.1009863)

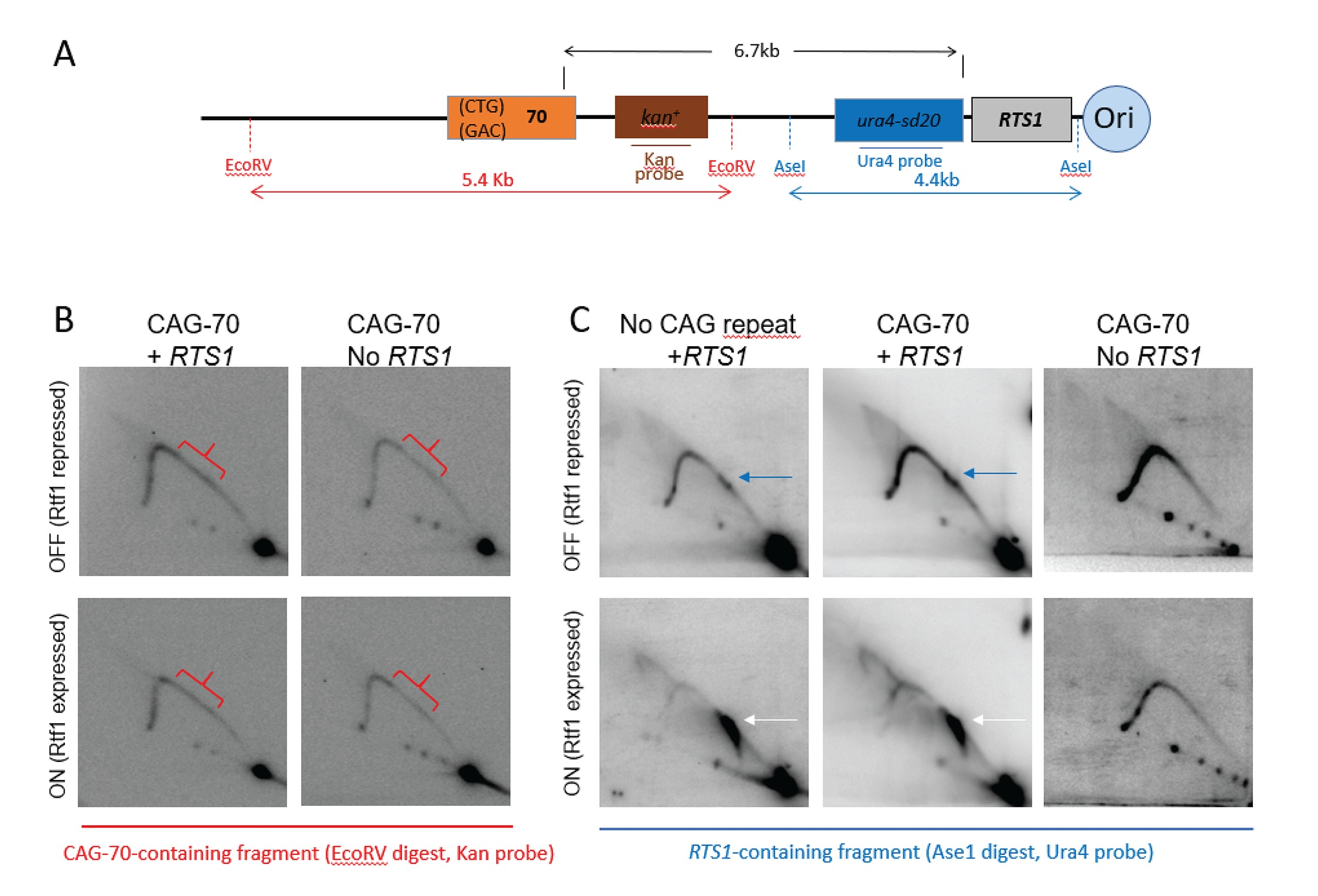

Supplement: S1 Fig — (A) Diagram of restriction fragments and probes used within the construct containing the CAG/CTG repeats integrated 6.7 kb downstream the RTS1-RFB. (B) examples of 2D gel analysis within the EcoRV restriction fragment containing the CAG-70 repeat tract in indicated strains and conditions (Off: Rtf1 is repressed, On: Rtf1 is expressed). Red brackets indicate the location of the CAG-70 repeats within the ascending arc. (C) Examples of 2D gel analysis within the AseI restriction fragment containing the RTS1-RFB in indicated strains and conditions (Off: Rtf1 is repressed, On: Rtf1 is expressed). Blue arrows indicate fork stalling at the RTS1-RFB. Weak fork stalling was detected when Rtf1 is repressed (referred to as Weak RFB condition) compared to the strain devoid of RTS1 sequence (top panels). A stronger stall (white arrows) was observed upon Rtf1 expression (Strong RFB condition). A signal for the converging fork can be seen coming off the top of the Y arc when the RFB is induced (see S1C Fig ON condition, hook-shaped signal, diagrammed in S3C Fig) (TIF) [file pgen.1009863.s001.tif]

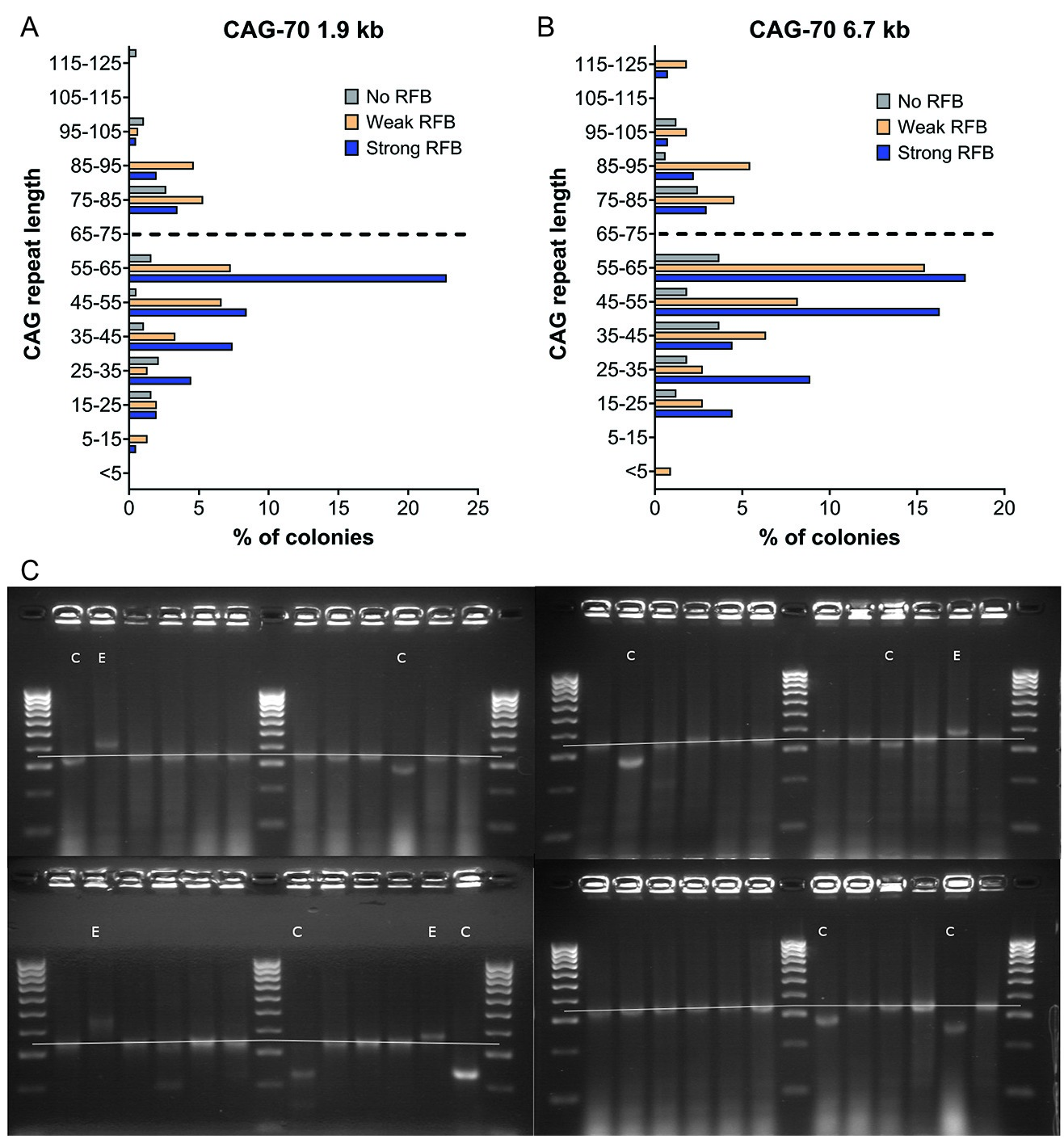

Supplement: S2 Fig — Analysis depicting CAG repeat contraction and expansion sizes for wild-type strains with a (CAG)70 starting tract size at the A) 1.9 kb and B) 6.7 kb locations across indicated conditions. Sizes were grouped in bins of 10 repeats (30 bp). Expansions were considered above 75 repeats and contractions below 65 repeats. A two sample KS statistical test showed significant differences in the repeat length distributions between conditions No vs. Weak RFB, No vs. Strong RFB, and Weak vs. Strong RFB for the 1.9 kb location; and No vs. Weak RFB and No vs. Strong RFB for 6.7 kb location (p < 0.005 for all. See Table M in S1 Text for a listing of the number of times each length was observed and p-values. (C) Four representative gels that were used to analyze the size of PCR products and determine their approximate size are shown. Bands above the drawn line were scored as expansions (E), and bands below the line as contractions (C). Lanes with more than one PCR product (intact and changed) were scored as an expansion or contraction if at least 50% of the signal intensity was contained in the band with an altered size. (TIF) [file pgen.1009863.s002.tif]

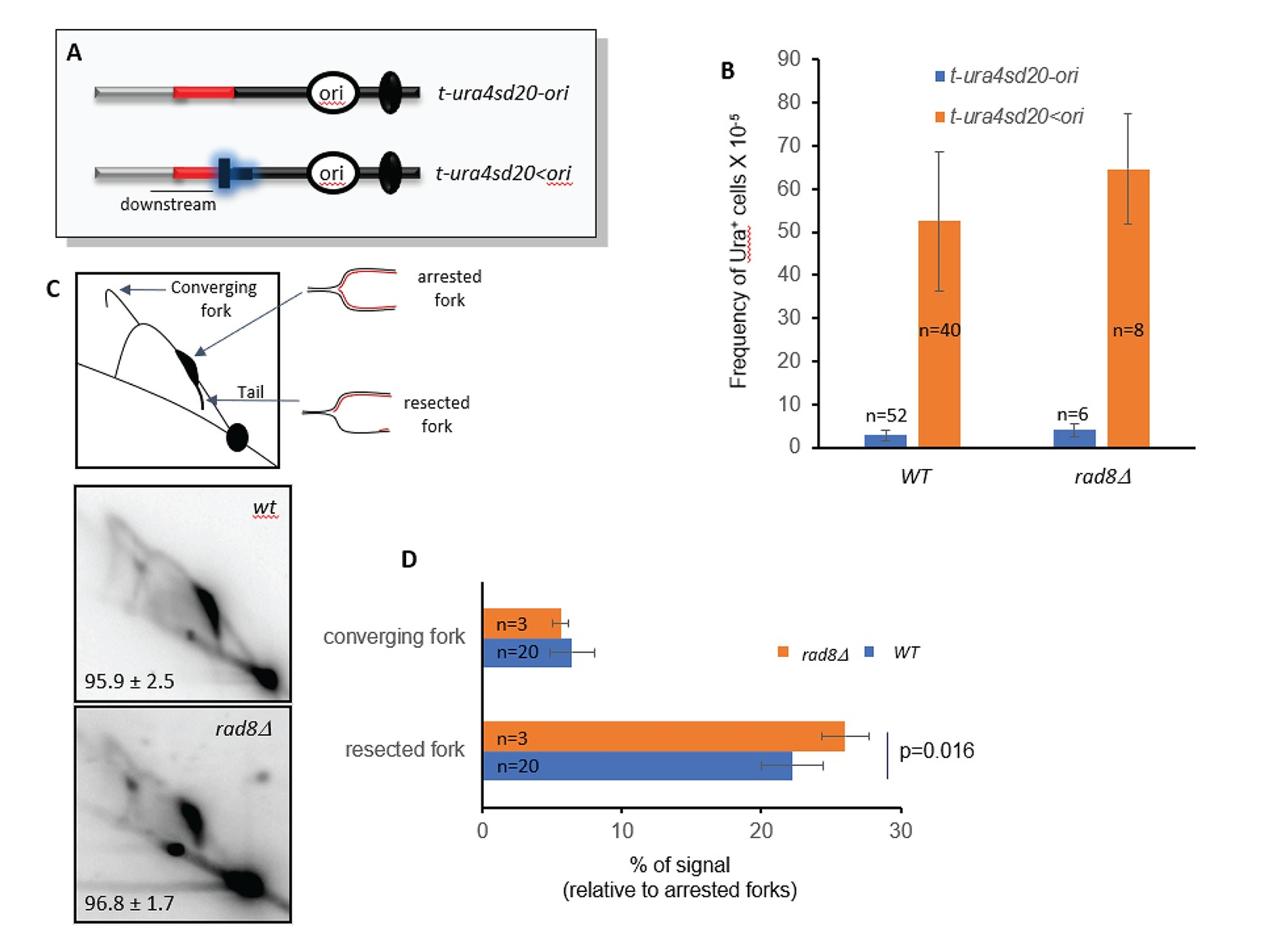

Supplement: S3 Fig — (A) Diagram of constructs containing the reporter allele ura4-sd20 (red bars) associated with the RFB (t-ura4sd20<ori) or not (t-ura4sd20-ori). The non-functional ura4-sd20 allele contains a 20-nt duplication flanked by micro-homology and is located downstream of the RFB. Upon activation of the RFB, ura4-sd20 is replicated by the restarted fork liable to replication slippage, resulting in the deletion of the duplication and restoring a functional ura4+ gene to generate Ura+ cells. As a control, the construct lacking the RTS1 sequence was used to monitor the spontaneous frequency of replication slippage with no RFB present. (B) Frequency of Ura+ cells in indicated strains. Values are means from n independent biological samples and error bars indicate standard deviation. (C) Top panel: Scheme of replication intermediates (RI) analyzed by neutral-neutral 2DGE of the AseI restriction fragment in RFB ON conditions, as described on S1 Fig. Signals corresponding to converging fork, arrested fork and resected fork (tail signal) are indicated [27,39]. Bottom panels: Representative RI analysis in indicated strains. The ura4 gene was used as probe. Numbers indicate the percentage of forks blocked by the RFB ± standard deviation. (D) Quantification of resected fork and converging fork in indicated strains. Values are means from n independent biological samples and error bars indicate standard deviation. p values were calculated using the non-parametric Mann Whitney test. (TIF) [file pgen.1009863.s003.tif]

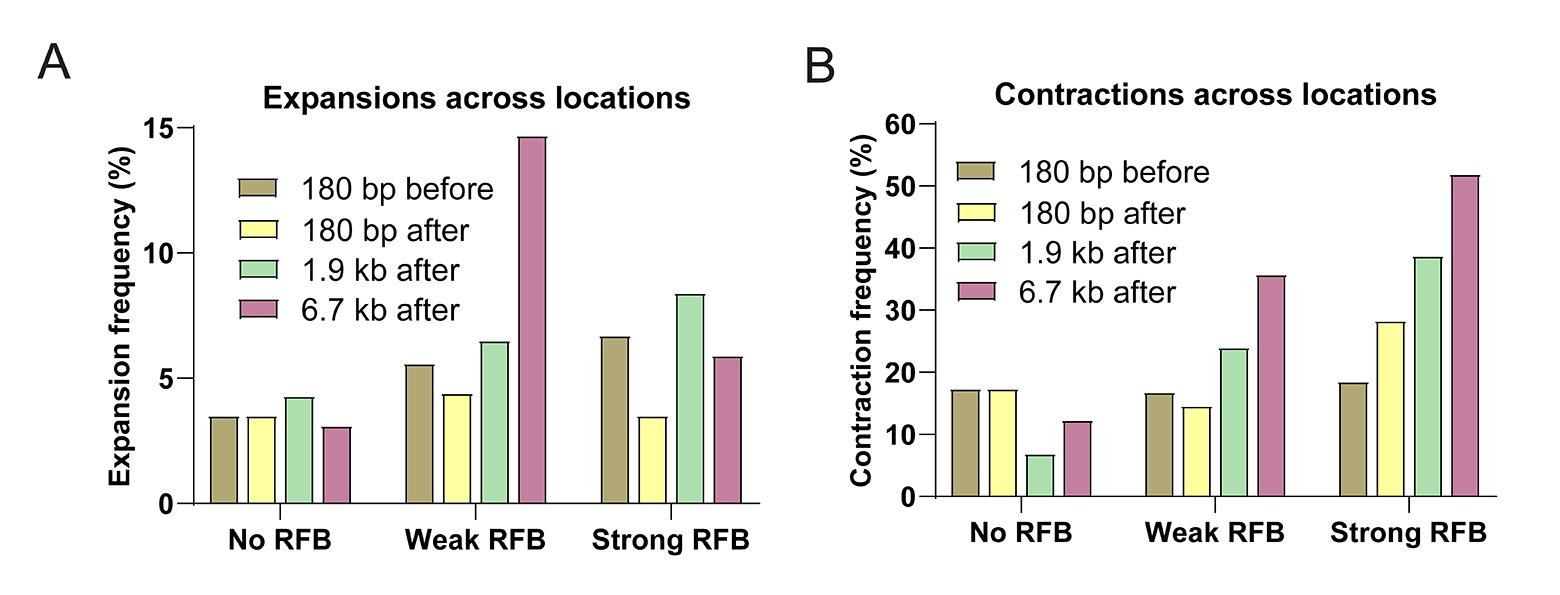

Supplement: S4 Fig — (A) Summary of frequency of CAG-70 expansions detected at the indicated locations. (B) Summary of frequency of CAG-70 contractions detected at the indicated locations. The CAG repeat is at the same location, replacing the RTS1 sequence (~ 5 kb from ori 3004/3005) in the No RFB 180 bp before and after locations, thus the instability data is the same for those two bars. (TIF) [file pgen.1009863.s004.tif]
