# Supplementary material for: Restarted replication forks are error-prone and cause CAG repeat expansions and contractions
Source: PLoS Genet. 2021 Oct 21;17(10):e1009863. doi: 10.1371/journal.pgen.1009863 (PMC8562783; doi:10.1371/journal.pgen.1009863)
Supplement: S1 Text — The S1 text contains Tables A through O. Tables A to J contain raw CAG instability data for all the strains tested. Tables K and L contain p values for instability comparisons between strains. Table M contains the CAG expansion and contraction size distributions for the wild-type strain. Table N contains the S. pombe strains used in this study. Table O contains the primers used in this study. (DOCX) [file pgen.1009863.s005.docx]

**S1 Text: Supplementary Tables**

**Table A: Wild-type CAG-70 at 1.9 kb and 6.7 kb raw instability data**

| **Strain** | **Location** | **Condition** | **Assay** | **Total # Colonies** | **Expansions** | | **Contractions** | |
| --- | --- | --- | --- | --- | --- | --- | --- | --- |
|  |  |  |  |  | **#** | **%** | **#** | **%** |
| Wild-type CAG-70 | 1.9 kb | No RFB | 1 | 95 | 3 | 3.2 | 6 | 6.3 |
|  |  |  | 2 | 93 | 5 | 5.4 | 7 | 7.5 |
|  |  |  | Combined | 188 | 8 | 4.3 | 13 | 6.9 |
|  |  | Weak RFB | 1 | 74 | 5 | 6.8 | 18 | 24.3 |
|  |  |  | 2 | 69 | 9 | 13.0 | 17 | 24.6 |
|  |  |  | 3 | 94 | 4 | 4.3 | 26 | 27.7 |
|  |  |  | 4 | 192 | 10 | 5.2 | 42 | 21.9 |
|  |  |  | Combined | 429 | 28 | 6.5 | 103 | 24.0 |
|  |  | Strong RFB | 1 | 149 | 8 | 5.4 | 72 | 48.3 |
|  |  |  | 2 | 52 | 4 | 7.7 | 19 | 36.5 |
|  |  |  | 3 | 94 | 5 | 5.3 | 39 | 41.5 |
|  |  |  | 4 | 192 | 24 | 12.5 | 59 | 30.7 |
|  |  |  | Combined | 487 | 41 | 8.4 | 189 | 38.8 |
|  | 6.7 kb | No RFB | 1 | 79 | 3 | 3.8 | 11 | 13.9 |
|  |  |  | 2 | 84 | 2 | 2.4 | 9 | 10.7 |
|  |  |  | Combined | 163 | 5 | 3.1 | 20 | 12.3 |
|  |  | Weak RFB | 1 | 41 | 3 | 7.3 | 18 | 43.9 |
|  |  |  | 2 | 68 | 13 | 19.1 | 21 | 30.9 |
|  |  |  | Combined | 109 | 16 | 14.7 | 39 | 35.8 |
|  |  | Strong RFB | 1 | 63 | 2 | 3.2 | 35 | 55.6 |
|  |  |  | 2 | 72 | 6 | 8.3 | 35 | 48.6 |
|  |  |  | Combined | 135 | 8 | 5.9 | 70 | 51.9 |

**Table B: *rad52Δ* CAG-70 at 1.9 kb and 6.7 kb raw instability data**

| **Strain** | **Location** | **Condition** | **Assay** | **Total # Colonies** | **Expansions** | | **Contractions** | |
| --- | --- | --- | --- | --- | --- | --- | --- | --- |
|  |  |  |  |  | **#** | **%** | **#** | **%** |
| *rad52Δ* CAG-70 | 1.9 kb | No RFB | 1 | 8 | 0 | 0.0 | 0 | 0.0 |
|  |  |  | 2 | 21 | 4 | 19.0 | 7 | 33.3 |
|  |  |  | 3 | 15 | 0 | 0.0 | 3 | 20.0 |
|  |  |  | 4 | 24 | 1 | 4.2 | 8 | 33.3 |
|  |  |  | 5 | 53 | 4 | 7.5 | 24 | 45.3 |
|  |  |  | 6 | 18 | 1 | 5.6 | 5 | 27.8 |
|  |  |  | Combined | 139 | 10 | 7.2 | 47 | 33.8 |
|  |  | Weak RFB | 1 | 41 | 0 | 0.0 | 13 | 31.7 |
|  |  |  | 2 | 22 | 0 | 0.0 | 9 | 40.9 |
|  |  |  | 3 | 54 | 0 | 0.0 | 20 | 37.0 |
|  |  |  | Combined | 117 | 0 | 0.0 | 42 | 35.9 |
|  |  | Strong RFB | 1 | 53 | 3 | 5.7 | 21 | 39.6 |
|  |  |  | 2 | 31 | 2 | 6.5 | 16 | 51.6 |
|  |  |  | 3 | 38 | 2 | 5.3 | 16 | 42.1 |
|  |  |  | Combined | 122 | 7 | 5.7 | 53 | 43.4 |
|  | 6.7 kb | No RFB | 1 | 12 | 2 | 16.7 | 2 | 16.7 |
|  |  |  | 2 | 22 | 0 | 0.0 | 13 | 59.1 |
|  |  |  | 3 | 79 | 10 | 12.7 | 23 | 29.1 |
|  |  |  | 4 | 15 | 6 | 40.0 | 5 | 33.3 |
|  |  |  | 5 | 27 | 0 | 0.0 | 13 | 48.1 |
|  |  |  | 6 | 83 | 6 | 7.2 | 34 | 41.0 |
|  |  |  | Combined | 238 | 24 | 10.1 | 90 | 37.8 |
|  |  | Weak RFB | 1 | 38 | 2 | 5.3 | 19 | 50.0 |
|  |  |  | 2 | 75 | 0 | 0.0 | 35 | 46.7 |
|  |  |  | Combined | 113 | 2 | 1.8 | 54 | 47.8 |
|  |  | Strong RFB | 1 | 40 | 1 | 2.5 | 16 | 40.0 |
|  |  |  | 2 | 69 | 3 | 4.3 | 48 | 69.6 |
|  |  |  | Combined | 109 | 4 | 3.7 | 64 | 58.7 |

**Table C: *rad8Δ* CAG-70 at 1.9 kb raw instability data**

| **Strain** | **Location** | **Condition** | **Assay** | **Total # Colonies** | **Expansions** | | **Contractions** | |
| --- | --- | --- | --- | --- | --- | --- | --- | --- |
|  |  |  |  |  | **#** | **%** | **#** | **%** |
| *rad8Δ* CAG-70 | 1.9 kb | No RFB | 1 | 58 | 7 | 12.1 | 9 | 15.5 |
|  |  |  | 2 | 118 | 15 | 12.7 | 30 | 25.4 |
|  |  |  | 3 | 44 | 1 | 2.3 | 6 | 13.6 |
|  |  |  | 4 | 22 | 2 | 9.1 | 5 | 22.7 |
|  |  |  | 5 | 81 | 3 | 3.7 | 21 | 25.9 |
|  |  |  | 6 | 44 | 4 | 9.1 | 4 | 9.1 |
|  |  |  | Combined | 367 | 32 | 8.7 | 75 | 20.4 |
|  |  | Weak RFB | 1 | 60 | 0 | 0.0 | 6 | 10.0 |
|  |  |  | 2 | 41 | 4 | 9.8 | 8 | 19.5 |
|  |  |  | Combined | 101 | 4 | 4.0 | 14 | 13.9 |
|  |  | Strong RFB | 1 | 50 | 2 | 4.0 | 16 | 32.0 |
|  |  |  | 2 | 35 | 1 | 2.9 | 13 | 37.1 |
|  |  |  | Combined | 85 | 3 | 3.5 | 29 | 34.1 |

**Table D: *swi10Δ* CAG-70 at 1.9 kb raw instability data**

| **Strain** | **Location** | **Condition** | **Assay** | **Total # Colonies** | **Expansions** | | **Contractions** | |
| --- | --- | --- | --- | --- | --- | --- | --- | --- |
|  |  |  |  |  | **#** | **%** | **#** | **%** |
| *swi10Δ* CAG-70 | 1.9 kb | No RFB | 1 | 27 | 1 | 3.7 | 1 | 3.7 |
|  |  |  | 2 | 15 | 2 | 13.3 | 2 | 13.3 |
|  |  |  | 3 | 48 | 1 | 2.1 | 7 | 14.6 |
|  |  |  | Combined | 90 | 4 | 4.4 | 10 | 11.1 |
|  |  | Weak RFB | 1 | 45 | 1 | 2.2 | 13 | 28.9 |
|  |  |  | 2 | 30 | 2 | 6.7 | 4 | 13.3 |
|  |  |  | Combined | 75 | 3 | 4.0 | 17 | 22.7 |
|  |  | Strong RFB | 1 | 28 | 1 | 3.6 | 11 | 39.3 |
|  |  |  | 2 | 37 | 7 | 18.9 | 16 | 43.2 |
|  |  |  | Combined | 65 | 8 | 12.3 | 27 | 41.5 |

**Table E: *exo1Δ* CAG-70 at 1.9 kb raw instability data**

| **Strain** | **Location** | **Condition** | **Assay** | **Total # Colonies** | **Expansions** | | **Contractions** | |
| --- | --- | --- | --- | --- | --- | --- | --- | --- |
|  |  |  |  |  | **#** | **%** | **#** | **%** |
| *exo1Δ* CAG-70 | 1.9 kb | No RFB | 1 | 48 | 4 | 8.3 | 8 | 16.7 |
|  |  |  | 2 | 48 | 2 | 4.2 | 7 | 14.6 |
|  |  |  | 3 | 47 | 2 | 4.3 | 3 | 6.4 |
|  |  |  | 4 | 46 | 1 | 2.2 | 7 | 15.2 |
|  |  |  | Combined | 189 | 9 | 4.8 | 25 | 13.2 |
|  |  | Weak RFB | 1 | 48 | 4 | 8.3 | 11 | 22.9 |
|  |  |  | 2 | 44 | 1 | 2.3 | 16 | 36.4 |
|  |  |  | 3 | 45 | 2 | 4.4 | 13 | 28.9 |
|  |  |  | Combined | 137 | 7 | 5.1 | 40 | 29.2 |
|  |  | Strong RFB | 1 | 47 | 7 | 14.9 | 18 | 38.3 |
|  |  |  | 2 | 37 | 1 | 2.7 | 12 | 32.4 |
|  |  |  | 3 | 44 | 1 | 2.3 | 20 | 45.5 |
|  |  |  | Combined | 128 | 9 | 7.0 | 50 | 39.1 |

**Table F: *rad52Δexo1Δ* CAG-70 at 1.9 kb raw instability data**

| **Strain** | **Location** | **Condition** | **Assay** | **Total # Colonies** | **Expansions** | | **Contractions** | |
| --- | --- | --- | --- | --- | --- | --- | --- | --- |
|  |  |  |  |  | **#** | **%** | **#** | **%** |
| *rad52*Δ *exo1Δ* CAG-70 | 1.9 kb | No RFB | Combined | 94 | 5 | 5.3 | 13 | 13.8 |
|  |  | Weak RFB | Combined | 143 | 1 | 0.7 | 42 | 29.4 |
|  |  | Strong RFB | Combined | 140 | 1 | 0.7 | 57 | 40.7 |

**Table G: *msh2Δ* CAG-70 at 1.9 kb raw instability data**

| **Strain** | **Location** | **Condition** | **Assay** | **Total # Colonies** | **Expansions** | | **Contractions** | |
| --- | --- | --- | --- | --- | --- | --- | --- | --- |
|  |  |  |  |  | **#** | **%** | **#** | **%** |
| *msh2Δ* CAG-70 | 1.9 kb | No RFB | 1 | 47 | 1 | 2.1 | 12 | 25.5 |
|  |  |  | 2 | 42 | 1 | 2.4 | 10 | 23.8 |
|  |  |  | 3 | 48 | 0 | 0.0 | 10 | 20.8 |
|  |  |  | 4 | 47 | 1 | 2.1 | 7 | 14.9 |
|  |  |  | Combined | 184 | 3 | 1.6 | 39 | 21.2 |
|  |  | Weak RFB | 1 | 30 | 0 | 0.0 | 5 | 16.7 |
|  |  |  | 2 | 60 | 3 | 5.0 | 28 | 46.7 |
|  |  |  | Combined | 90 | 3 | 3.3 | 33 | 36.7 |
|  |  | Strong RFB | 1 | 41 | 1 | 2.4 | 15 | 36.6 |
|  |  |  | 2 | 59 | 3 | 5.1 | 24 | 40.7 |
|  |  |  | Combined | 100 | 4 | 4.0 | 39 | 39.0 |

**Table H: Wild-type CTG-70 at 1.9 kb raw instability data**

| **Strain** | **Location** | **Condition** | **Assay** | **Total # Colonies** | **Expansions** | | **Contractions** | |
| --- | --- | --- | --- | --- | --- | --- | --- | --- |
|  |  |  |  |  | **#** | **%** | **#** | **%** |
| Wild type CTG-70 | 1.9 kb | No RFB | Combined | 96 | 1 | 1 | 52 | 54.2 |
|  |  | Weak RFB | Combined | 143 | 1 | 0.7 | 108 | 75.5 |
|  |  | Strong RFB | Combined | 144 | 7 | 4.9 | 98 | 68.1 |

**Table I: CAG-70 at 3.0 kb, plus 13 Terminators raw instability data**

| **Strain** | **Location** | **Condition** | **Assay** | **Total # Colonies** | **Expansions** | | **Contractions** | |
| --- | --- | --- | --- | --- | --- | --- | --- | --- |
|  |  |  |  |  | **#** | **%** | **#** | **%** |
| 13 Terminators CTG-70 | 3.0 kb | Weak RFB | Combined | 186 | 9.0 | 4.8 | 40 | 21.5 |
|  |  | Strong RFB | Combined | 109 | 7.0 | 6.4 | 26 | 23.9 |

**Table J: Wild-type CAG-70 at 180 bp Before or After RFB raw instability data**

| **Strain** | **Location** | **Condition** | **Assay** | **Total # Colonies** | **Expansions** | | **Contractions** | |
| --- | --- | --- | --- | --- | --- | --- | --- | --- |
|  |  |  |  |  | **#** | **%** | **#** | **%** |
| Wild-type CAG-70 | 180 bp After RFB | No RFB | 1 | 19 | 0 | 0.0 | 1 | 5.3 |
|  |  |  | 2 | 16 | 1 | 6.3 | 3 | 18.8 |
|  |  |  | 3 | 21 | 1 | 4.8 | 4 | 19.0 |
|  |  |  | 4 | 21 | 1 | 4.8 | 3 | 14.3 |
|  |  |  | 5 | 17 | 0 | 0.0 | 7 | 41.2 |
|  |  |  | 6 | 21 | 1 | 4.8 | 2 | 9.5 |
|  |  |  | Combined | 115 | 4 | 3.5 | 20 | 17.4 |
|  |  | Weak RFB | 1 | 41 | 1 | 2.4 | 9 | 22.0 |
|  |  |  | 2 | 48 | 3 | 6.3 | 5 | 10.4 |
|  |  |  | 3 | 48 | 2 | 4.2 | 6 | 12.5 |
|  |  |  | Combined | 137 | 6 | 4.4 | 20 | 14.6 |
|  |  | Strong RFB | 1 | 45 | 3 | 6.7 | 11 | 24.4 |
|  |  |  | 2 | 21 | 0 | 0.0 | 5 | 23.8 |
|  |  |  | 3 | 47 | 1 | 2.1 | 16 | 34.0 |
|  |  |  | Combined | 113 | 4 | 3.5 | 32 | 28.3 |
|  | 180 bp Before RFB | No RFB | 1 | 19 | 0 | 0.0 | 1 | 5.3 |
|  |  |  | 2 | 16 | 1 | 6.3 | 3 | 18.8 |
|  |  |  | 3 | 21 | 1 | 4.8 | 4 | 19.0 |
|  |  |  | 4 | 21 | 1 | 4.8 | 3 | 14.3 |
|  |  |  | 5 | 17 | 0 | 0.0 | 7 | 41.2 |
|  |  |  | 6 | 21 | 1 | 4.8 | 2 | 9.5 |
|  |  |  | Combined | 115 | 4 | 3.5 | 20 | 17.4 |
|  |  |  | 1 | 48 | 2 | 4.2 | 10 | 20.8 |
|  |  | Weak RFB | 2 | 47 | 3 | 6.4 | 4 | 8.5 |
|  |  |  | 3 | 48 | 3 | 6.3 | 10 | 20.8 |
|  |  |  | Combined | 143 | 8 | 5.6 | 24 | 16.8 |
|  |  | Strong RFB | 1 | 48 | 7 | 14.6 | 7 | 14.6 |
|  |  |  | 2 | 41 | 1 | 2.4 | 8 | 19.5 |
|  |  |  | 3 | 46 | 1 | 2.2 | 10 | 21.7 |
|  |  |  | Combined | 135 | 9 | 6.7 | 25 | 18.5 |

**Table K: p-values for wild-type and mutant strain instability comparisons**

| **Strain** | **Location** | **Condition** | **Expansions** | | | **Contractions** | | |
| --- | --- | --- | --- | --- | --- | --- | --- | --- |
|  |  |  | **p-value to WT same condition and location** | **p-value to No RFB** | **p-value to Weak RFB** | **p-value to WT same condition and location** | **p-value to No RFB** | **p-value to Weak RFB** |
| **Wild-type CAG-70** | 1.9 kb | No RFB | NA | NA | 0.35 | NA | NA | 0.0001 |
|  |  | Weak RFB | NA | 0.35 | NA | NA | 0.0001 | NA |
|  |  | Strong RFB | NA | 0.07 | 0.31 | NA | 0.0001 | 0.0001 |
|  | 6.7 kb | No RFB | NA | NA | 0.0008 | NA | NA | 0.0001 |
|  |  | Weak RFB | NA | 0.0008 | NA | NA | 0.0001 | NA |
|  |  | Strong RFB | NA | 0.26 | 0.03 | NA | 0.0001 | 0.01 |
| ***rad52Δ*** | 1.9 kb | No RFB | 0.33 | NA | 0.002 | 0.0001 | NA | 0.79 |
|  |  | Weak RFB | 0.002 | 0.002 | NA | 0.01 | 0.79 | NA |
|  |  | Strong RFB | 0.45 | 0.8 | 0.01 | 0.35 | 0.13 | 0.24 |
|  | 6.7 kb | No RFB | 0.01 | NA | 0.004 | 0.05 | NA | 0.08 |
|  |  | Weak RFB | 0.0004 | 0.004 | NA | 0.08 | 0.08 | NA |
|  |  | Strong RFB | 0.56 | 0.05 | 0.44 | 0.3 | 0.0003 | 0.11 |
| ***rad8Δ*** | 1.9 kb | No RFB | 0.06 | NA | 0.14 | 0.0001 | NA | 0.15 |
|  |  | Weak RFB | 0.49 | 0.14 | NA | 0.03 | 0.15 | NA |
|  |  | Strong RFB | 0.18 | 0.12 | 1 | 0.47 | 0.01 | 0.002 |
| ***swi10Δ*** | 1.9 kb | No RFB | 1 | NA | 1 | 0.36 | NA | 0.06 |
|  |  | Weak RFB | 0.6 | 1 | NA | 0.88 | 0.06 | NA |
|  |  | Strong RFB | 0.35 | 0.12 | 0.02 | 0.69 | 0.0001 | 0.02 |
| ***exo1Δ*** | 1.9 kb | No RFB | 1 | NA | 1 | 0.06 | NA | 0.0004 |
|  |  | Weak RFB | 0.68 | 1 | NA | 0.26 | 0.0004 | NA |
|  |  | Strong RFB | 0.72 | 0.46 | 0.61 | 1 | 0.0001 | 0.09 |
| ***rad52*Δ *exo1Δ*** | 1.9 kb | No RFB | 0.77 | NA | 0.04 | 0.08 | NA | 0.007 |
|  |  | Weak RFB | 0.004 | 0.04 | NA | 0.22 | 0.007 | NA |
|  |  | Strong RFB | 0.0004 | 0.04 | 1 | 0.7 | 0.0001 | 0.05 |
| ***msh2Δ*** | 1.9 kb | No RFB | 0.22 | NA | 0.398 | 0.0001 | NA | 0.008 |
|  |  | Weak RFB | 0.33 | 0.398 | NA | 0.02 | 0.008 | NA |
|  |  | Strong RFB | 0.15 | 0.25 | 1 | 1 | 0.002 | 0.77 |

Gray highlight: no RFB values significant between wt and mutant (indicating mutant effect independent of RFB). Yellow highlight: values significant between the RFB (either weak or strong) conditions and no RFB, indicating a RFB stall-specific effect; no RFB to weak RFB comparisons that appear twice in the table are only highlighted once. Green highlight: RFB values significant between wt and mutant (indicating mutant effect in RFB condition).

**Table L: p-values for strains with repeats integrated at different locations**

| **Strain** | **Location** | **Condition** | **Expansions** | | | | **Contractions** | | | |
| --- | --- | --- | --- | --- | --- | --- | --- | --- | --- | --- |
|  |  |  | **p-value to CAG-70 at**  **1.9 kb** | **p-value to CAG-70 at**  **6.7 kb** | **p-value to No RFB** | **p-value to Weak RFB** | **p-value to CAG-70 at**  **1.9 kb** | **p-value to CAG-70 at**  **6.7 kb** | **p-value to No RFB** | **p-value to Weak RFB** |
| **Wild-type CAG-70** | 1.9 kb | No RFB | NA | 0.587 | NA | 0.35 | NA | 0.14 | NA | 0.0001 |
|  |  | Weak RFB | NA | 0.009 | 0.35 | NA | NA | 0.02 | 0.0001 | NA |
|  |  | Strong RFB | NA | 0.47 | 0.07 | 0.31 | NA | 0.11 | 0.0001 | 0.0001 |
|  | 6.7 kb | No RFB | 0.587 | NA | NA | 0.0008 | 0.14 | NA | NA | 0.0001 |
|  |  | Weak RFB | 0.009 | NA | 0.0008 | NA | 0.02 | NA | 0.0001 | NA |
|  |  | Strong RFB | 0.47 | NA | 0.26 | 0.03 | 0.11 | NA | 0.0001 | 0.01 |
| **Wild-type CTG-70** | 1.9 kb | No RFB | 0.28 | 0.42 | NA | 1 | 0.0001 | 0.0001 | NA | 0.0007 |
|  |  | Weak RFB | 0.004 | 0.009 | 1 | NA | 0.0001 | 0.0001 | 0.0007 | NA |
|  |  | Strong RFB | 0.21 | 0.79 | 0.15 | 0.07 | 0.0001 | 0.007 | 0.04 | 0.19 |
| **13 Ter** | 3.0 kb | Weak RFB | 0.47 | 0.005 | NA | NA | 0.53 | 0.01 | NA | NA |
|  |  | Strong RFB | 0.56 | 1 | NA | 0.6 | 0.004 | 0.0001 | NA | 0.08 |
| **Wild-type CAG-70** | 180 bp After | No RFB | 1 | 1 | NA | 0.76 | 0.002 | 0.29 | NA | 0.07 |
|  |  | Weak RFB | 0.42 | 0.006 | 0.76 | NA | 0.02 | 0.0002 | 0.61 | NA |
|  |  | Strong RFB | 0.11 | 0.55 | 1 | 1 | 0.04 | 0.0002 | 0.06 | 0.01 |
| **Wild-type CAG-70** | 180 bp Before | No RFB | 1 | 1 | NA | 0.56 | 0.007 | 0.29 | NA | 1 |
|  |  | Weak RFB | 0.84 | 0.02 | 0.56 | NA | 0.08 | 0.0007 | 1 | NA |
|  |  | Strong RFB | 0.59 | 1 | 0.39 | 0.8 | 0.0001 | 0.0001 | 0.87 | 0.75 |

Gray highlight: no RFB values significant between CAG-70 at 1.9 kb and other location listed (indicating effect independent of RFB). Yellow highlight: values significant between the RFB (either weak or strong) conditions and no RFB, or between the weak and strong RFBs, indicating a RFB stall-specific effect; no RFB to weak RFB comparisons that appear twice in the table are only highlighted once. Green highlight:

| **Location** | **Condition** | **CAG Repeat Length (total # colonies)** | | | | | | | | | | | | | | **Total # Colonies** | |  | |
| --- | --- | --- | --- | --- | --- | --- | --- | --- | --- | --- | --- | --- | --- | --- | --- | --- | --- | --- | --- |
|  |  | **<5** | **5-15** | **15-25** | **25-35** | **35-45** | **45-55** | **55-65** | **65-75** | **75-85** | **85-95** | **95-105** | **105-115** | **115-125** |  | |  | |  |
| 1.9 kb | No RFB | 0 | 0 | 3 | 4 | 2 | 1 | 3 | 167 | 5 | 0 | 2 | 0 | 1 | 188 | |  | |  |
|  | Weak RFB | 0 | 2 | 3 | 2 | 5 | 10 | 11 | 102 | 8 | 7 | 1 | 0 | 0 | 151 | |  | |  |
|  | Strong RFB | 0 | 1 | 4 | 9 | 15 | 17 | 46 | 98 | 7 | 4 | 1 | 0 | 0 | 202 | |  | |  |
| 6.7 kb | No RFB | 0 | 0 | 2 | 3 | 6 | 3 | 6 | 136 | 4 | 1 | 2 | 0 | 0 | 163 | |  | |  |
|  | Weak RFB | 1 | 0 | 3 | 3 | 7 | 9 | 17 | 55 | 5 | 6 | 2 | 0 | 2 | 110 | |  | |  |
|  | Strong RFB | 0 | 0 | 6 | 12 | 6 | 22 | 24 | 56 | 4 | 3 | 1 | 0 | 1 | 135 | |  | |  |
| **Location** | **Condition** | **CAG Repeat Length (% of total # colonies)** | | | | | | | | | | | | | | **p-value to No RFB*** | | **p-value to Weak RFB*** | |
|  |  | **<5** | **5-15** | **15-25** | **25-35** | **35-45** | **45-55** | **55-65** | **65-75** | **75-85** | **85-95** | **95-105** | **105-115** | **115-125** |  | |  | |  |
| 1.9 kb | No RFB | 0.0 | 0.0 | 1.6 | 2.1 | 1.1 | 0.5 | 1.6 | 88.8 | 2.7 | 0.0 | 1.1 | 0.0 | 0.5 | NA | | 0.0023 | |  |
|  | Weak RFB | 0.0 | 1.3 | 2.0 | 1.3 | 3.3 | 6.6 | 7.3 | 67.5 | 5.3 | 4.6 | 0.7 | 0.0 | 0.0 | 0.0023 | | NA | |  |
|  | Strong RFB | 0.0 | 0.5 | 2.0 | 4.5 | 7.4 | 8.4 | 22.8 | 48.5 | 3.5 | 2.0 | 0.5 | 0.0 | 0.0 | <0.0001 | | 0.0009 | |  |
| 6.7 kb | No RFB | 0.0 | 0.0 | 1.2 | 1.8 | 3.7 | 1.8 | 3.7 | 83.4 | 2.5 | 0.6 | 1.2 | 0.0 | 0.0 | NA | | <0.0001 | |  |
|  | Weak RFB | 0.9 | 0.0 | 2.7 | 2.7 | 6.4 | 8.2 | 15.5 | 50.0 | 4.5 | 5.5 | 1.8 | 0.0 | 1.8 | <0.0001 | | NA | |  |
|  | Strong RFB | 0.0 | 0.0 | 4.4 | 8.9 | 4.4 | 16.3 | 17.8 | 41.5 | 3.0 | 2.2 | 0.7 | 0.0 | 0.7 | <0.0001 | | 0.0535 | |  |

RFB values significant between CAG-70 at 1.9 kb or 6.7 kb and other location as listed (indicating effect in RFB condition).

**Table M. Expansion and contraction size distribution for wild-type CAG-70 strains**

*p-values were calculated using the Kolmogorov-Smirnov Test.

**Table N. *S. pombe* strains used in the RFB Fork Restart Assays. RFB designated as “<” in the strain nomenclature, which indicates the polarity of the RFB.**

| **CFP Number** | **Name** | **Genotype** |
| --- | --- | --- |
| 3  4  149 | CAG70@1.9 kb<RTS1-ori | *h-, t-CAG70-kanMX6@ura4sd20<ori, rtf1:nmt41:sup35, ade6-704, leu1-32* |
| 11  12  13 | CAG70@6.7 kb<RTS1-ori | *h-, t-CAG70-kanMX6@4.8kb-ura4sd20<ori, rtf1:nmt41:sup35, ade6-704, leu1-32* |
| 17  19 | CAG70@1.9 kb-ori | *h-, t-CAG70-kanMX6@ura4sd20-ori, rtf1:nmt41:sup35, ade6-704, leu1-32* |
| 23  24 | CAG70@6.7 kb-ori, *rad52Δ* | *h+, rad52::natMX6, t-CAG70-kanMX6@4.8kb-ura4sd20-ori, rtf1:nmt41:sup35, ade6-704, leu1-32* |
| 25 | CAG70@6.7 kb<RTS1-ori, *rad52Δ* | *h+, rad52::natMX6, t-CAG70-kanMX6@4.8kb-ura4sd20<ori, rtf1:nmt41:sup35, ade6-704, leu1-32* |
| 27  29 | CAG70@1.9 kb-ori, *rad52Δ* | *h+, rad52::natMX6, t-CAG70-kanMX6@ura4sd20-ori, rtf1:nmt41:sup35, ade6-704, leu1-32* |
| 30  31 | CAG70@1.9 kb<RTS1-ori, *rad52Δ* | *h+, rad52::natMX6, t-CAG70-kanMX6@ura4sd20<ori, rtf1:nmt41:sup35, ade6-704 leu1-32* |
| 37  38 | CAG70@1.9 kb-ori, *rad8Δ* | *h-, rad8::hphMX6, t-CAG70-kanMX6@ura4sd20-ori, rtf1:nmt41:sup35, ade6-704, leu1-32* |
| 39  40 | CAG70@1.9 kb<RTS1-ori, *rad8Δ* | *h-, rad8::hphMX6, t-CAG70-kanMX6@ura4sd20<ori, rtf1:nmt41:sup35, ade6-704, leu1-32* |
| 47 | CAG70@1.9 kb-ori, *msh2Δ* | *h+, msh2::kanMX6, t-kanMX6-CAG70@ura4sd20-ori, rtf1:nmt41:sup35, ade6-704, leu1-32* |
| 49  50 | CAG70@1.9 kb<RTS1-ori, *msh2Δ* | *h+, msh2::kanMX6, t-kanMX6-CAG70@ura4sd20<ori, rtf1:nmt41:sup35, ade6-704, leu1-32* |
| 50 | CAG70@1.9 kb<RTS1-ori, *msh2Δ* | *h+, msh2::kanMX6, t-kanMX6-CAG70@ura4sd20<ori, rtf1:nmt41:sup35, ade6-704, leu1-32* |
| 65  66 | 13Ter-CAG70@3.0 kb<RTS1-ori | *h-, t-13XTer-kanMX6-CAG70-ura4sd20-ura5<ori, ura5::hphMX6, rtf1:nmt41:sup35, ade6-704, leu1-32* |
| 71  72 | RTS1<180 bp-CAG70-ori (180 bp before RFB) | *ura4::CAG70, t-AscI<ura4-AscI-ori, rtf1:nmt41:sup35, ade6-704, leu1-32* |
| 75 | CAG70@180 bp<RTS1-ori (180 bp after RFB) | *ura4::CAG130* (contracted to CAG73), *t-AscI-ura4<AscI-ori, rtf1:nmt41:sup35, ade6-704, leu1-32* |
| 76 | CAG70@180 bp<RTS1-ori (180 bp after RFB) | *ura4::CAG130* (contracted to CAG90, contracted to CAG73), *t-AscI-ura4<AscI-ori, rtf1:nmt41:sup35, ade6-704, leu1-32* |
| 81  82 | CAG70@1.9 kb-ori, *swi10Δ* | *h+, swi10::kanMX6, t-kanMX6-CAG70@ura4sd20-ori, rft1:nmt41:sup35, ade6-704, leu1-32* |
| 84  85 | CAG70@1.9 kb<RTS1-ori, *swi10Δ* | *h+, swi10::kanMX6, t-kanMX6-CAG70@ura4sd20<ori, rtf1:nmt41:sup35, ade6-704, leu1-32* |
| 86 | CAG70@1.9 kb<RTS1-ori, *swi10Δ* | *h-, swi10::kanMX6, t-kanMX6-CAG70@ura4sd20<ori, rtf1:nmt41:sup35, ade6-704, leu1-32* |
| 90  91 | CAG70@1.9 kb-ori, *exo1Δ* | *h-, exo1::natMX6, t-kanMX6-CAG70@ura4sd20-ori, rtf1:nmt41:sup35, ade6-704, leu+* |
| 92  93  94 | CAG70@1.9 kb<RTS1-ori, exo1Δ | *h- or h+, exo1::natMX6, t-kanMX6-CAG70@ura4sd20<ori, rtf1:nmt41:sup35, ade6-704, leu+* |
| 95 | CAG70@1.9 kb-ori, *rad52Δ* ,*exo1*Δ | *h-, rad52::natMX6, exo1::natMX6, t-kanMX6-CAG70@ura4sd20-ori, rtf1:nmt41:sup35, ade6-704, leu+* |
| 97 | CAG70@1.9 kb<RTS1-ori, *rad52Δ,* *exo1Δ* | *h-, rad52::natMX6, exo1::natMX6, t-kanMX6-CAG70@ura4sd20<ori, rtf1:nmt41:sup35, ade6-704, leu+* |
| 109  110  111 | CAG70-ori (180 bp No RTS1) | *h-, ura4::CAG70, rtf1:nmt41:sup35, ade6-704, leu1-32* |
| 124  125  127 | 13Ter-CAG70@3.0 kb-ori | *h-, t-13xTer-kanMX6-CAG70-ura4sd20-ura5-ori, ura5::hphMX6, rtf1:nmt41:sup35, ade6-704, leu1-32* |
| 137  138 | CTG@1.9 kb<RTS-ori | *h-, t-kanMX6-CTG70@ura4sd20<RTS1-ori, rtf1:nmt41:sup35, ade6-704, leu1-32* |

**Table O: Primer sequences used in this study**

| **Primer name** | **Sequence (5’ to 3’)** |
| --- | --- |
| CTGrev2 | CCCAGGCCTCCAGTTTGC |
| T720-B | GAATTCGAGCTCCACCGCGG |
| KF124 | ACGTTTTTTTCTTAAGAACATGTGATTGGAGCAATTTTAAAACCTATTTGCACCG ATATTGTGTATTATACTCCGAGAAAAAGTATACTAGTTTTGAATAAAACTGGAT GGCGGCGTTAG |
| KF125 | ATACCATGTAGACAAACAAGATAAAACTTGGTTATAAACATTGGTGTTGGAAC AGAATAAATTAGATGTCAAAAAGTTTCGTCAATATCACAAGCTTTATCCCAGGC CTCCAGTTTGC |
| KF130 | TGATATTGACGAAACTTTTTGACATCTAATTTATTCTGTTCCAACACCAATGTTTA TAACCAAGTTTTATCTTGTTTGTCTACATGGTATTTTACATTCAAAACTGGATGGC GGCGTTAG |
| KF131 | ACAATCTTTTCTCTTGGATTGACATTGAATAAGAAAAGAGTGAATTTTTTTAGAC TTGTAATGATAACTATGTACAAAGCCAATGAAAGATGTATGTAGACCCAGGCCT CCAGTTTGC |
